# A review of the *Bovis Calculus*’s intervention mechanism and clinical application in ischemic stroke

**DOI:** 10.3389/fphar.2024.1510779

**Published:** 2025-01-15

**Authors:** Ke Xu, Bowen Deng, Tongtong Jia, Mihong Ren, Hai Chen, Jing Zhang, Jinlin Guo, Yong Li, Jian Wang

**Affiliations:** College of Pharmacy, Chengdu University of Traditional Chinese Medicine, Chengdu, China

**Keywords:** *Bovis Calculus*, chemical composition, ischemia stroke, clinical application, drug safety, pharmacokinetics, traditional application, pharmacological effect

## Abstract

**Background:**

*Bovis Calculus* (BC), also named Niuhuang in Chinese, is utilized as a resuscitation drug in Traditional Chinese Medicine (TCM) for the treatment of neurological disorders. Ischemic stroke (IS) is a significant global public health issue that currently lacks safe and effective therapeutic drugs. Ongoing efforts are focused on identifying effective treatment strategies from Traditional, Complementary, and Integrative Medicine. Noticeably, BC has been used in TCM for thousands of years to prevent or treat IS-related diseases.

**Methods:**

The historical origins of BC in the treatment of IS were investigated through the examination of ancient Chinese medical texts. Furthermore, the chemical components of BC were analyzed, and its mechanisms of action against IS were summarized using literature sourced from databases such as Web of Science, PubMed, and China National Knowledge Infrastructure. Information on Chinese medicine preparations and clinical reports was also integrated to provide an overview of modern applications and safety considerations.

**Results:**

BC mainly includes chemical components such as bile pigments, bile acids, cholesterol, proteins amino acids, and trace elements. Additionally, the efficacy of BC in treating cerebral ischemia/reperfusion injury (CI/RI) is certain, particularly due to the components of bile pigments, bile acids, and amino acids that can interfere with the enzymatic cascade reaction of CI/RI through multiple components, targets, and pathways. The active components of BC exert neuroprotective effects by reducing microcirculation disturbance, excitatory amino acid toxicity, and oxidative stress injury in the acute stage; inhibiting inflammatory injury, apoptosis, and blood-brain barrier (BBB) disruption in the subacute stage; and promoting angiogenesis and neurogenesis in the restoration stage. Furthermore, as a crude drug, BC appears in many Chinese patent medicine (CPM) preparations for the treatment of IS, and clinical and preclinical studies have proved its safety.

**Conclusion:**

The use of BC in the treatment of IS has a long history, proven efficacy, and widespread application. Future efforts should focus on elucidating its mechanisms of action and exploring its applications.

## Graphical Abstract

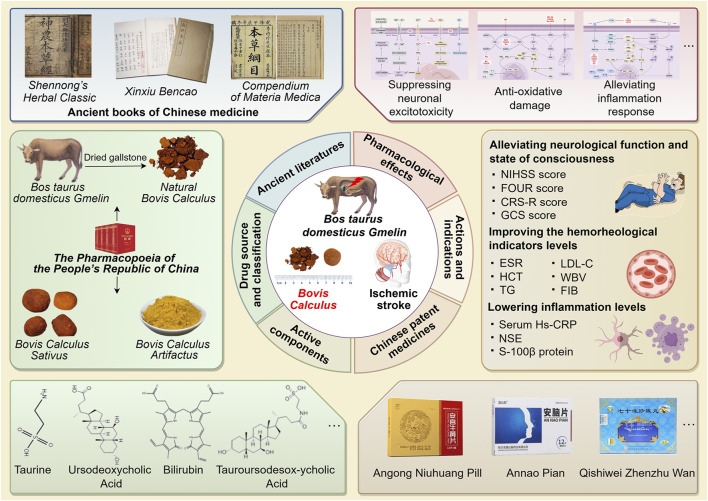

## 1 Introduction

*Bovis Calculus* (BC) is the desiccated gallstone of *Bos taurus domesticus* Gmelin. It has been employed for cardio-cerebrovascular ailment treatment for over two millennia in China. According to the *Chinese Pharmacopoeia*, this substance is described as having a cooling and sweet nature and is associated with the heart and liver meridians. It is known for its ability to cleanse the heart, eliminate phlegm, induce resuscitation, cool the liver, relieve wind-related symptoms, and detoxify the body. Thus, people use BC to treat stroke and phlegm confusion, fever and dizziness, epileptic convulsions, etc. (Chinese Pharmacopoeia Committee of People’s Republic of China, 2020). Due to the scarce source and high price of natural BC, nowadays, *Bovis Calculus Artifactus* and *Bovis Calculus Sativus* have been studied as alternatives to natural BC.

Stroke is a severe disease of cerebral blood circulation disturbance and a major public health event that results in death and disability on a global scale. It is caused by cerebral vascular obstruction or rupture, leading to damage to tissue function and structure. With its high mortality and disability rates, stroke has brought a heavy burden to society and is now the second most deadly disease in the world (Owolabi et al., 2015; Johnson et al., 2016; Wu et al., 2019). It is noteworthy that CI is the primary type of stroke, accounting for about 85% of all strokes (Sarvari et al., 2020). Studies found that CI/RI is the result of the interaction of multiple pathological components such as microcirculation disturbance, energy metabolism disorder, oxidative stress, inflammatory responses, apoptosis, and necrosis (Sekerdag et al., 2018; Przykaza, 2021). Nowadays, thrombolysis with recombinant tissue-type plasminogen activator (rt-PA) is the main approach to restoring cerebral blood flow (CBF). However, thrombolysis is subject to a strict time window. The use of rt-PA also increases the risk of intracranial hemorrhage, potentially converting CI to cerebral hemorrhage (Cheng et al., 2014; Emberson et al., 2014). Therefore, it is imperative to determine the ideal treatment drug.

As studies showed, the active components of BC that reduce the acute stage of CI/RI are taurine (Tau), ursodeoxycholic acid (UDCA), bilirubin (BR), biliverdin (BV), cholic acid (CA), tauroursodeoxycholic acid (TUDCA) and hyodeoxycholic acid (HDCA). Besides, the active components of BC that inhibit the subacute stage of CI/RI are CA, UDCA, TUDCA, HDCA, Tau, BV, BR and glycoursodeoxycholic acid (GUDCA). Otherwise, the active components of BC that promote the restoration stage of cerebral ischemic recovery are Tau, CA and HDCA.

Up to now, there have been numerous pharmacological studies on the treatment of IS by BC and its active components. The effectiveness and mechanism of BC has also been extensively validated in animal studies (Wang et al., 2014; Zhang et al., 2021). However, there is a lack of a review to systematically sort out the relevant content. This study not only seeks to combine three fundamental sources of BC and its chemical constituents to investigate the molecular mechanism through which BC influences CI/RI, but also discussed its widely clinical application.

## 2 Materials and methods

A comprehensive search was conducted on the medical databases, including Web of Science, PubMed, China National Knowledge Infrastructure (CNKI), Wanfang Data, and VIP Database, using terms such as “*Bovis Calculus*” “natural *Bovis Calculus*” “*Bovis Calculus* artifactus” “*Bovis Calculus* sativus” “cerebral ischemia” “ischemic stroke” “traditional applications” “chemical components” “clinical applications” “clinical safety” “pharmacokinetic characteristics” “safety evaluation”. The languages of retrieval include English and non-English. The types of literature include journals, papers, books, and patents. A total of 376 articles were retrieved. Then the retrieved literature was categorized and summarized. Exclusion criteria included literature with poor quality, old data and lack of innovation. And the search time frame is from 1 January 1955, to 31 August 2024. Finally, 196 relevant papers were obtained. There were 112 English papers and 84 non-English papers, and the ratio of English to non-English papers was approximately 1.33:1. All the papers included in this manuscript can fully support the conclusions of this manuscript.

## 3 Traditional applications of *Bovis Calculus*

The traditional applications of BC can be traced back to *Shennong’s Herbal Classic* (Wang and Xue, 2008). For over two thousand years, its effectiveness and recommended uses have evolved, from *Shennong’s Herbal Classic* to the latest edition of the *Chinese Pharmacopoeia* (Chen and Wang, 2023). Earlier studies have indicated that BC can be used as follows, clear the heart, sweep phlegm, open the neorifices, cool the liver, extinguish wind, and remove toxin, indications loss of consciousness in febrile disease, wind-stroke and phlegm clouding the heart, seizures and convulsions, epilepsy, manic psychosis, swollen sore throat, mouth and tongue sores, swelling abscess, deep-rooted boil and sore.

In the periods of the *Qin* and *Han* Dynasties (221 B.C. to 220 A.D.), *Shennong’s Herbal Classic* recorded that BC was mainly used for convulsive epilepsy, aversion to cold first and then fever, hyperpyrexia, syncope, and spasm. It was the first time to record the application of BC in infantile convulsions, febrile convulsions, and mental and emotional diseases. After that, the efficacy and indications of BC in the *Collection of herbal classics* (Tao, 1955) in the *Liang* Dynasty (502 A.D. to 557 A.D.) and the *Xinxiu Bencao* (Su et al., 2004) in the *Tang* Dynasty (618 A.D. to 907 A.D.) were consistent. Such as treatment for children with various diseases including epilepsy, excessive internal heat leading to mouth failure to open, and adult mania. Later, the *Five* Dynasties (907 A.D. to 979 A.D.) *Rihuazi herbal medicine* (Ri and Shang, 2005): treatment of apoplectic aphasia, lockjaw, palpitations, infectious disease, forgetfulness, and debility, increased the records of BC in stroke and infectious diseases treatment. Hundred years later, in the *Yuan* Dynasty (1271 A.D. to 1368 A.D.), the record of diuretic phlegm and anticonvulsants was added to the *Daily Materia medica* (Wu, 2019). Specifically, in the *Ming* Dynasty (1368 A.D. to 1644 A.D.), Li Shizhen’s *Compendium of Materia Medica* (Li et al., 2014) recorded that BC could be used for children with febrile convulsions, chewing tongue, and delirium.

## 4 Chemical components of *Bovis Calculus*

**FIGURE 1:**
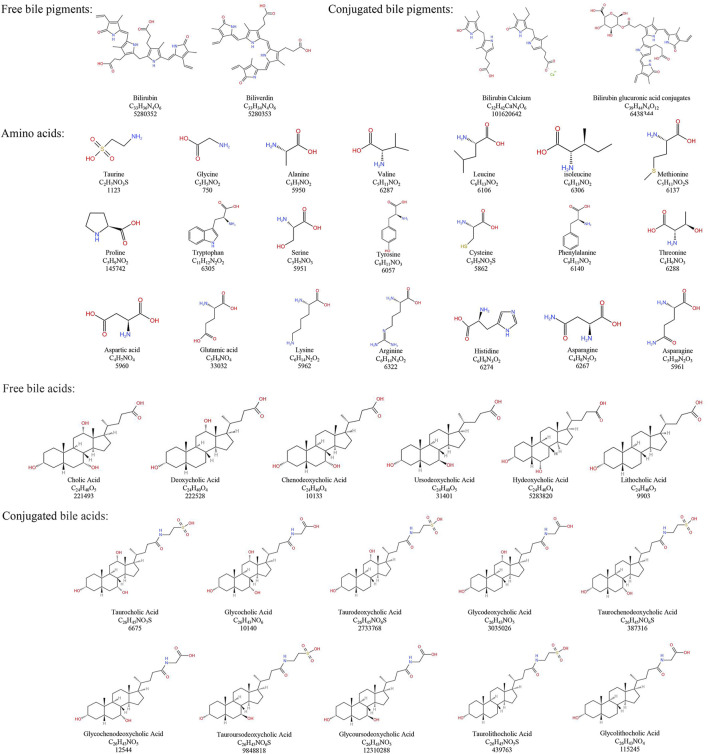
Chemical structure of bile pigments, amino acids, and bile acids in *Bovis Calculus*.

Modern research has elucidated the chemical composition of NBC more clearly. However, due to the scarcity and high price of NBC, in recent years, *Bovis Calculus Artifactus* (BCA), *Bovis Calculus Sativus* (BCS), cultured *Bovis Calculus* (CBC), and other substitutes for NBC have been successfully developed to address urgent clinical needs (Yang et al., 1996). It is noteworthy that the BC discussed in this paper does not pertain to the CBC and is consistent with medical ethics. Besides, the chemical composition and content of BC from different base sources differ (Kong et al., 2010; 2012; Yu et al., 2020). The chemical composition of NBC and its common clinical substitutes along with their structures, are sorted out as follows (Table 1; Figure 1).

**TABLE 1:** The main chemical composition of BC.

Classification	Chemical component	Molecular formula	PubChem CID	Reference
Bile pigments	Bilirubin	C_33_H_36_N_4_O_6_	5280352	Jia et al. (2013), Miao et al. (2013), Feng et al. (2017), Huang et al. (2018a), Liu et al. (2019b), Yu et al. (2020)
	Bilirubin calcium	C_32_H_42_CaN_4_O_6_	101620642	
	Bilirubin glucuronic acid conjugates	C_39_H_44_N_4_O_12_	6438344	
	Biliverdin	C_33_H_34_N_4_O_6_	5280353	
Bile acids	Cholic acid	C_24_H_40_O_5_	221493	
	Deoxycholic acid	C_24_H_40_O_4_	222528	
	Chenodeoxycholic acid	C_24_H_40_O_4_	10133	
	Ursodeoxycholic acid	C_24_H_40_O_5_	31401	
	Hydeoxycholic acid	C_24_H_40_O_4_	5283820	
	Lithocholic acid	C_24_H_40_O_3_	9903	
	Taurocholic acid	C_26_H_45_NO_7_S	6675	
	Glycocholic acid	C_26_H_43_NO_6_	10140	
	Taurodeoxycholic acid	C_26_H_45_NO_6_S	2733768	
	Glycodeoxycholic acid	C_26_H_43_NO_5_	3035026	
	Taurochenodeoxycholic acid	C_26_H_45_NO_6_S	387316	
	Glycochenodeoxycholic acid	C_26_H_43_NO_5_	12544	
	Tauroursodeoxycholic acid	C_26_H_45_NO_6_S	9848818	
	Glycoursodeoxycholic acid	C_26_H_43_NO_5_	12310288	
	Taurolithocholic acid	C_26_H_45_NO_5_S	439763	
	Glycolithocholic acid	C_26_H_43_NO_4_	115245	
Proteins and amino acids	Taurine	C_2_H_7_NO_3_S	1123	
	Glycine	C_2_H_5_NO_2_	750	
	Alanine	C_3_H_7_NO_2_	5950	
	Valine	C_5_H_11_NO_2_	6287	
	Leucine	C_6_H_13_NO_2_	6106	
	Isoleucine	C_6_H_13_NO_2_	6306	
	Methionine	C_5_H_11_NO_2_S	6137	
	Proline	C_5_H_9_NO_2_	145742	
	Tryptophan	C_11_H_12_N_2_O_2_	6305	
	Serine	C_3_H_7_NO_3_	5951	
	Tyrosine	C_9_H_11_NO_3_	6057	
	Cysteine	C_3_H_7_NO_2_S	5862	
	Phenylalanine	C_9_H_11_NO_2_	6140	
	Threonine	C_4_H_9_NO_3_	6288	
	Aspartic acid	C_4_H_7_NO_4_	5940	
	Glutamic acid	C_5_H_9_NO_4_	33032	
	Lysine	C_6_H_14_N_2_O_2_	5962	
	Arginine	C_6_H_14_N_4_O_2_	6322	
	Histidine	C_6_H_9_N_3_O_2_	6274	
	Asparagine	C_4_H_8_N_2_O_3_	6267	
	Glutamine	C_5_H_10_N_2_O_3_	5961	
Cholestenes	Ergosterol	C_28_H_44_O	444679	
	Cholesteryl ester	C_45_H_76_O_3_	90471597	
	Cholesterol	C_27_H_46_O	5997	
Others	Vitamin D3	C_27_H_44_O	5280795	
	Phosphatidylcholine	C_44_H_84_NO_8_P	6441487	
	Oleanic acid	C_30_H_48_O_3_	10494	
	Ursolic acid	C_30_H_48_O_3_	64945	
	Methyl cholate	C_25_H_42_O_5_	10960835	
	Carotenoid B	C_40_H_58_O	14730337	
Microelement	K, Na, Ca, Mg, Fe, P, Mn, Cu, Cl, Co, Ti, Cr, Ni, Pb, Zn, Ba, La, Li, Mo, Al, V, Sr and Y			

### 4.1 Natural *Bovis Calculus*

Natural *Bovis Calculus* (NBC) mainly contains bile pigments, bile acids, cholesterol, peptides, and inorganic elements (Kong et al., 2012). Among them, Bile pigments mainly exist in the form of conjugated BR and also contain a small amount of free BR and BV. Conjugated BR has been found to be one of the important active components of BC (Kong et al., 2010). Besides, Bile acid is one of the main medicinal components in NBC. Additionally, Bile acids in NBC are categorized as free bile acids and conjugated bile acids. Researches found that the free bile acids are mainly CA, deoxycholic acid (DCA), chenodeoxycholic acid (CDCA), UDCA, HDCA, and lithocholic acid. Another studies showed that the conjugated bile acids are mainly taurocholic acid (TCA), glycocholic acid (GCA), taurodeoxycholic acid (TDCA), glycodeoxycholic acid (GDCA), taurochenodeoxycholic acid (TCDCA), glycochenodeoxycholic acid (GCDCA), TUDCA, GUDCA, taurolithocholic acid and glycolithocholic acid (Kong et al., 2012; Yu et al., 2020). Tau is also one of the important active components in NBC (Kong et al., 2012). Furthermore, NBC contains components such as vitamin D and carotenoids (Kong et al., 2010). According to the *Pharmacopoeia of the People’s Republic of China,* NBC must contain not only a minimum of 4.0% bile acid but also at least 25% BR in dried form (Chinese Pharmacopoeia Committee of People’s Republic of China, 2020).

### 4.2 *Bovis Calculus* artifactus

The *Bovis Calculus Artifactus* (BCA) process is based on the known composition of NBC. Earlier studies have indicated that BCA consists of bovine bile powder, CA, DCA, Tau, BR, cholesterol, calcium phosphate, magnesium sulfate, and other trace elements (Kong et al., 2010; 2012). Compared to NBC, BCA has a higher concentration of total bile acids, but a lower level of BR (Yan et al., 2007). Noticeably, BCA and NBC share similar physical and chemical properties, but their chemical components’ content and ratio differ. As a result, there may be some differences in clinical efficacy compared to NBC. The *Pharmacopoeia of the People’s Republic of China* therefore specifies that BCA must contain a minimum of 13.0% bile acid and at least 0.63% BR in dried form (Chinese Pharmacopoeia Committee of People’s Republic of China, 2020).

### 4.3 Bovis Calculus sativus

*Bovis Calculus Sativus* (BCS) is artificially cultivated using fresh bile from cattle as the mother liquor. It involves the addition of CA, DCA, and other compounds to mimic the process of stone formation in cattle *in vitro* through physical and biochemical pathways. According to researches, it has similar physical and chemical properties to NBC and has similar clinical efficacy. However, the BR and BV content of BCS is lower than that of NBC. And the total bile acid content is higher than that of NBC (Cai et al., 2004; Wan et al., 2009). *The Pharmacopoeia of the People’s Republic of China* stipulates that BCS must contain not only a minimum of 6.0% bile acid but also at least 35% BR in dried form (Chinese Pharmacopoeia Committee of People’s Republic of China, 2020).

In addition, it has been demonstrated that the bound component of BC drugs has better efficacy compared to the free component. For instance, the NBC contains more bound bile acids and bound bile pigments, which may be one of the reasons for its better efficacy (Xu et al., 2017).

### 4.4 Comparison of chemical components of three types BC

The experiments demonstrated that Tau and conjugated bile acids content in BCS and NBC was significantly higher compared to the content in BCA (Feng et al., 2015). There was a significant difference in bile acids content between NBC and its two alternatives, BCS and BCA (Kong et al., 2012). Researchers utilized the Hierarchical Cluster Analysis (HCA) to categorize NBC into three groups, A, B, and C, based on quality. They also assessed conjugated bile acids and free bile acids levels in BCS and the various NBC groups. The results indicated higher levels of conjugated bile acids in BCS and group A (High-quality NBC), and higher levels of free bile acids in group B (Common NBC). High-quality NBC is similar to BCS in bile acids (Li et al., 2022). Additionally, study revealed that the total content of six bile acids in 33 batches of NBC samples ranged from 0 to 245.89 mg/g, while the total bile acids content in 2 batches of BCA samples ranged from 178.48 to 194.22 mg/g, and 3 batches of BCS samples ranged from 41.01 to 107.3 mg/g (Kong et al., 2012).

Furthermore, TCA content in NBC is significantly higher compared to the content of TCA in BCA. The content of GCA in BCA is notably higher than in BCS, while the content of GCA in NBC is relatively lower (Shimada et al., 2013). Utilizing liquid chromatography coupled with triple quadrupole mass spectrometry, researchers measured the levels of various bile acids in NBC, with GCA being the highest at 46%, followed by TCA at 25%, GDCA at 17%, TDCA at 10%, and GCDCA, GHDCA, and TCDCA each below 1%. In addition, in BCA, the content of CA reached 35%, TCA at 19%, DCA at 11%, HDCA at 10%, TDCA at 9%, GCA at 9%, and GDCA and CDCA both at 3% (Qiao et al., 2011). Chromatographic analysis also revealed that in BCA, the content of CA, CDCA, DCA, UDCA, TCDCA, and TDCA exceeded 9.88 μg/mg, while the bile acids content in NBC was lower (Ye et al., 2016). Due to commercial factors, there were significant variations in the three types of BC bile acids content, with bile acids content generally lower in NBC compared to BCS and BCA; it is possible that BCA may have higher bile acids content than NBC.

Additionally, the two alternatives to NBC, BCA and BCS, contain higher amounts of Tau, CA, Fe, Mg, and Ca. These components can serve as markers to differentiate NBC from its alternatives (Shimada et al., 2013). Researchers identified a new chemical compound in BCS named 3α, 12α-dihydroxy-7-oxo-5α-cholanic acid, which was scarcely detected in NBC and BCA. Furthermore, glycocholic acid, glycodeoxycholic acid, and taurocholic acid (TCA) were detected as biomarkers in NBC and BCA (Liu et al., 2015).

In conclusion, BCS is uniformly produced by pharmaceutical companies, with stable chemical composition. BCA is synthesized from various raw materials in specific proportions, resulting in higher purity and uniform chemical composition. On the other hand, NBC has a complex origin closely linked to the cattle’s environment, leading to significant variations in chemical composition among different batches.

## 5 Mechanisms of *Bovis Calculus* against CI/RI

The progression of CI/RI can be categorized into acute, subacute, and restoration stages. BC and its active constituents exhibit therapeutic effects on various aspects of CI/RI, with their mechanisms of action elucidated below (Table 2).

**TABLE 2:** Summary of anti-cerebral ischemia injury experiment of *Bovis Calculus*.

Drugs	Animal models and methods	Results	Reference
Taurine	1. Animals: SD rats (♂), 280–340 g 2. Model: MCAO 3. Ischemia time: Unclear 4. Route of administration: iv 5. Drug treating times: 4 h after MCAO 6. Administration dosage: 50 mg/kg	1. Tau treatment reduced cerebral infarction volume in rats 2. Tau treatment reduced the permeability of BBB in rats 3. Tau treatment almost completely abolished gelatinolytic activity in ischemic brain microvessels 4. Tau treatment significantly inhibited the increase of MMP-9 activity induced by CI 5. Tau treatment significantly inhibited the expression of CD147 protein induced by CI 6. Tau blocks tPA-related bleeding by inhibiting CD147 - dependent MMP-9 pathway in ischemic brain endothelial cells after IS	Jin et al. (2018)
Taurine	1. Animals: SD rats (♂), 250–280 g 2. Model: tMCAO 3. Ischemia time: 2 h 4. Route of administration: ip 5. Drug treating times: 48 h, 24 h and 2 h before MCAO, 0.5 h and 12 h after MCAO 6. Administration dosage: 200 mg/kg	1. Tau treatment reduced neurological deficit score in rats 2. Tau treatment reduced the volume of cerebral infarction in rats 3. Tau pretreatment reduced brain water content in rats 4. Tau (600 mg/kg ig) treatment reduces mean blood pressure and heart rate in conscious and anesthetized rats 5. Tau treatment can significantly restore the number of neurons in hippocampus and cortex after MCAO 6. Tau inhibits the expression of caspase-3 in cerebral infarction area	Wang et al. (2007a)
TUDCA	1. Animals: SD rats (♂), 180–200 g 2. Model: tMCAO 3. Ischemia time: 2 h 4. Route of administration: iv 5. Drug treating times: 1 h after modeling 6. Administration dosage: 400 mg/kg	1. TUDCA treatment decreased the level of Glu 2. TUDCA treatment remarkably improved the degree of cerebral infarction in ACI rats 3. TUDCA treatment improved the blood lipid level in ACI rats 4. TUDCA treatment inhibited mRNA and protein expressions of TNF-α, IL-8 and hs-CRP and alleviated inflammatory response 5. TUDCA treatment inhibited the expression of MDA and ox-LDL, increased the expression of SOD and GSH-Px, and alleviated the ACI-induced oxidative stress injury 6. TUDCA reduced the expression of VLDLR and NF-κB proteins and downregulated the expression of Bax and caspase-3 through negative regulation of Nrf2 signaling pathway	Bian et al. (2019)
Taurine	1. Animals: SD rats 2. Model: MCAO 3. Ischemia time: Unclear 4. Route of administration: iv 5. Drug treating times: 1 h after CI 6. Administration dosage: 50 mg mL^-1^·kg^-1^	1. Tau treatment significantly reduced the number of microglia containing MHC I antigen in rat CI area 2. Tau treatment significantly reduced the number of MHC II antigen positive microglia in cerebral ischemic area of rats 3. Tau inhibited NF-κB activity in ischemic penumbra of rats 4. Tau significantly inhibited TNF-α activity in ischemic penumbra of rats	Wang and Xu (2017)
CA + HDCA	1. Animals: SD rats (♂), 310–350 g 2. Model: pMCAO 3. Ischemia time: permanent 4. Route of administration: ip 5. Drug treating times: 1 h before MCAO 6. Administration dosage: 21 mg/kg	1. CA + HDCA treatment reduced infarct volume in CI rats 2. CA + HDCA treatment downregulated the expression of TNF-α in CI rats 3. CA + HDCA treatment inhibited IL-1β level in CI rats 4. CA + HDCA treatment reduced endothelial cell injury and plasma vWF concentration in CI rats 5. CA + HDCA treatment reduced neuronal damage and serum NSE level in rats with CI	Hua et al. (2009)
CA	1. Animals: Newborn SD rats 2. Model: *in vitro* NVU 3. Ischemia time: 1 h 4. Route of administration: coculture 5. Drug treating times: 24 h before OGD/R, 1 h under OGD, 24 h after OGD/R 6. Administration dosage: 93.75 μg/mL, 11.72 μg/mL	1. CA treatment improved the BBB function remarkably 2. CA has a significant protective effect on the BBB characteristics and neurons in the NVU after OGD/R 3. CA treatment inhibited the expression of IL-1β, IL-6 and TNF-α 4. CA pretreatment fully reversed OGD/R induced cell apoptosis in neurons	Li et al. (2020b)
HDCA	1. Animals: SD rat pups 2. Model: *in vitro* NVU 3. Ischemia time: 1 h 4. Route of administration: coculture 5. Drug treating times: 24 h before OGD/R, 1 h under OGD 6. Administration dosage: 10.16 μg/mL, 2.54 μg/mL	1. HDCA treatment protected the cell in a dose-dependent manner 2. HDCA treatment markedly improved the integrity of the BBB following OGD/R 3. HDCA treatment significantly inhibited the release of TNF-α, IL-1βand IL-6 4. HDCA treatment significantly lower the apoptosis cells of OGD/R 5. HDCA treatment increased GDNF and BDNF expression and secretion levels in a dose-dependent manner	Li et al. (2019a)
Taurine	1. Animals: SD rats (♂), 300–320 g 2. Model: tMCAO 3. Ischemia time: Unclear 4. Route of administration: iv 5. Drug treating times: 1 h after MCAO 6. Administration dosage: 50 mg mL^-1^·kg^-1^	1. Tau treatment reduced the number of MHC I antigen positive microglia in ischemic gray matter, corpus callosum and caudate putamen of rats with CI 2. Tau treatment reduced the number of MHC II antigen positive microglia in ischemic gray matter, corpus callosum and caudate putamen of rats with CI	Wang et al. (2018)
Taurine	1. Animals: SD rats (♂), 315–340 g 2. Model: tMCAO 3. Ischemia time: 2 h 4. Route of administration: iv 5. Drug treating times: 2 h after MCAO 6. Administration dosage: 50 mg/kg	1. Tau treatment decreased the infarct volume and lessened the brain swelling significantly 2. Tau treatment markedly reduced the PARP levels in the cytosolic fractions in the core and in the nuclear fractions in the penumbra and core 3. Tau treatment suppressed the induction and activation of NF-κB in the penumbra and core after experimental stroke 4. Tau treatment markedly reduced the protein levels of TNF-α, IL-1β and ICAM-1 in the penumbra and core 5. Tau treatment markedly reduced the number of neutrophils in the penumbra and core 6. Tau treatment significantly reduced the cell death scores in the penumbra and core	Sun et al. (2012)
Taurine	1. Animals: SD rats (♂), 200–250 g 2. Model: pMCAO 3. Ischemia time: permanent 4. Route of administration: ip 5. Drug treating times: 1 week before MCAO 6. Administration dosage: 250 mg/kg	1. Tau pretreatment significantly improved cerebral infarction volume in rats with CI 2. Tau pretreatment inhibited the decrease of SOD activity after ischemia for 6 h 3. Tau pretreatment inhibited ICAM-1 expression in CI rats	Luo et al. (2004)
Taurine	1. Animals: Wistar rats, 250–300 g 2. Model: tMCAO 3. Ischemia time: 1 h 4. Route of administration: iv 5. Drug treating times: 30 min before MCAO 6. Administration dosage: 200 mg/kg	1. Tau pretreatment reduced cerebral infarction volume in rats with CI 2. Tau pretreatment reduced the swelling volume of ischemic brain tissue in ischemic rats 3. Tau pretreatment inhibits the expression of NF-κB in CI rats	Yang et al. (2006)
Biliverdin	1. Animals: SD rats (♂), 200–240 g 2. Model: tMCAO 3. Ischemia time: 2 h 4. Route of administration: ip 5. Drug treating times: 15 min before reperfusion, 4 h after reperfusion 6. Administration dosage: 35 mg/kg in 2 mL	1. BV treatment markedly reduced the NSS of rats 2. BV treatment markedly reduced the infarction 3. BV treatment significantly downregulated the mRNA expression level of HO-1 4. BV treatment significantly decreased the mRNA expression of TNF-α, IL-1β, iNOS and IL-6	Li et al. (2017)
Biliverdin	1. Animals: 1). SD rats (♂), 200–240 g, 2). SD rats pups 2. Model: 1). tMCAO, 2). in vitro hippocampal neurons I/R injury 3. Ischemia time: 2 h 4. Route of administration: 1). ip, 2). coculture 5. Drug treating times: 15 min before reperfusion, 4 h、12 h and 20 h after reperfusion 6. Administration dosage: 1). 35 mg/kg, 2). 2 μg/mL	1. BV treatment remarkedly suppressed the mRNA and protein expression levels of IL-6, IL-1β and TNF-α 2. BV treatment remarkably enhanced cell viability following OGD/R injury 3. BV treatment remarkably suppress the expression of caspase-3 and Bax 4. BV treatment promoted the expression of Bcl-2	Li et al. (2021)
Bilirubin	1. Animals: SD rats (♂), 280–300 g 2. Model: tMCAO 3. Ischemia time: 2 h 4. Route of administration: ip 5. Drug treating times: 2 h after reperfusion 6. Administration dosage: 2 mmol/L	1. Apoptosis rate, MDA, 8-OHdG, Bax and caspase-3 levels in ischemic penumbra decreased significantly after BR treatment in rats with CI 2. Expression of SOD, GSH and Bcl-2 in ischemic penumbra after BR treatment in rats with CI	Zhang et al. (2016)
Biliverdin	1. Animals: Wistar rats (♂), 250–250 g 2. Model: tMCAO 3. Ischemia time: 1.5 h 4. Route of administration: ip 5. Drug treating times: Immediately after reperfusion 6. Administration dosage: 100 mg/kg	1. BV treatment rapidly increased the serum levels of BR in a dose-dependent 2. BV treatment significantly deceased the infarct area in the cerebral cortices 3. BV treatment prevented superoxide generation following tMCAO 4. BV treatment significantly decreased the number of stained cells for these oxidative injury markers in the cortex 5. BV treatment significantly reduced 4-HNE expressions	Deguchi et al. (2008)
BCS	1. Animals: SD rats (♂), 240–280 g 2. Model: tMCAO 3. Ischemia time: 1.5 h 4. Route of administration: ig 5. Drug treating times: pre-dose for 3 days, 1 h before MCAO, 6 h after MCAO 6. Administration dosage: 50 mg/kg, 100 mg/kg	1. BCS treatment has a protective effect on CI/RI 2. BCS treatment inhibited neuronal apoptosis in response to CI/RI 3. BCS treatment alleviated the mitochondrial damage by CI/RI	Lu et al. (2020)

Abbreviations: ig, intragastric administration; ip: intraperitoneal injection; iv: intravenous injection; pMCAO, permanent middle cerebral artery occlusion; SD-rats: Sprague-Dawley rats; tMCAO, transient middle cerebral artery occlusion; Tau, Taurine; BV, biliverdin; CA, cholic acid; TUDCA, tauroursodeoxycholic acid; BR, bilirubin; BCS, *bovis calculus sativus;* BCA, *bovis calculus artifactus;* NBC, natural *bovis calculus;* BBB, Blood-brain barrier; MMP-9, Matrix metalloproteinase-9; Glu, Glutamic acid; TNF-α, Tumor necrosis factor-α; hs-CRP, High-sensitivity c-reactive protein; MDA, malondialdehyde; SOD, superoxide dismutase; ROS, reactive oxygen species; GSH, glutathione; Bax, Bax, Bcl-2, associated X protein; NF-κB, Nuclear factor-κb; LDL-C, Low-density lipoprotein cholesterol; MCP-1, Monocyte chemoattractant protein-1; IL-1β, Interleukin-1β; NVU, neurovascular unit; OGD/R, Oxygen-glucose deprivation/reperfusion; GDNF, Glia cell line-derived neurotrophic factor; BDNF, Brain-derived neurotrophic factor; iNOS, inducible nitric oxide synthase; IS, Ischemic stroke.

### 5.1 Mechanisms of against CI/RI in the acute stage

During the acute stage, reduced CBF leads to an insufficient supply of glucose and oxygen, resulting in unmet energy requirements for neurons, glial cells, and endothelial cells. Depolarization and reduced glutamate reuptake activity due to hypoxia leads to elevated extracellular glutamate levels. Elevated glutamate levels can cause intracellular calcium overload via N-methyl-D-aspartate ionotropic receptors (NMDAR) and metabotropic glutamate receptors.

#### 5.1.1 Improving microcirculation disturbances

The optimal treatments for CI involve administering thrombolysis promptly within the treatment time frame, and quickly and efficiently rebuilding microvascular collateral circulation. Reinstating blood reperfusion in the ischemic region and penumbra is also a critical intervention. If CBF is not restored in time, microcirculation abnormalities in the ischemic core and penumbra will be closely associated with inflammatory cell infiltration, microthrombosis, vascular endothelial cell damage, and other variables. Cerebral edema resulting from IS exacerbates microcirculatory disruptions by compressing capillaries, underscoring the importance of minimizing microcirculatory disturbances post-ischemia. Furthermore, enhancing CBF in the ischemic penumbra is equally crucial during the acute phase of CI (Kulik et al., 2008; Przykaza, 2021).

Tau is a crucial active ingredient in BC that increases CBF in cerebral ischemic tissue (Zhou et al., 2004; Wang et al., 2016). Following CI, the interaction between extracellular matrix metalloproteinase inducer and platelet glycoprotein VI can lead to intravascular fibrin deposition and platelet adhesion, and subsequent microthrombus formation (Seizer et al., 2009; Jin et al., 2017). According to research, Tau helps reduce secondary thrombosis of microvessels post-CI, aiding in facilitating reperfusion. Furthermore, Tau can mitigate the risk of hemorrhagic transformation following recombinant tissue-type plasminogen activator (rt-PA) treatment by inhibiting the extracellular matrix protease-inducible factor-dependent matrix metalloproteinase-9 (MMP-9) pathway in cerebral tissue post-ischemia (Jin et al., 2018).

Earlier studies have indicated that platelet aggregation is a significant contributor to thrombosis. Tau can stimulate the release of endogenous arachidonic acid (AA) triggered by collagen and inhibit the synthesis of thromboxane A2 (Chen et al., 1991). Tau also demonstrates a notable inhibitory impact on platelet aggregation, which is induced by adenosine diphosphate (ADP), collagen, and AA. In addition, the combination of Tau with tetrandrine or neferine has the potential to further suppress platelet aggregation, which is induced by ADP, collagen, and AA (Huang and Rao, 1995; Qi and Rao, 1995). Hypertension, a major risk factor for CI, has a complex interplay with microcirculatory disturbances, contributing to endothelial dysfunction and hypoperfusion due to increased shear stress (Feihl et al., 2009; Cipolla et al., 2018). In patients with essential hypertension, Tau can lower blood pressure. Moreover, Tau helps to prevent the increase of serum Ca^2+^ in spontaneously hypertensive rats on a high-salt diet, leading to reduced blood pressure (Dawson Jr et al., 2000; Militante and Lombardini, 2002; El Idrissi et al., 2013).

When combined with indapamide, Tau has the ability to significantly reduce blood pressure in spontaneously hypertensive rats and also shorten the duration of the dosing cycle. The mechanism is to affect the intracellular Ca^2+^ concentration, then promote the phosphorylation of endothelium-derived nitric oxide (NO) synthase, and increase the release of NO. Thus, leading to blood vessel relaxation and a reduction in blood pressure (Zhao Y. Y. et al., 2018). Research has also revealed that Tau exerts a non-endothelium-dependent vasodilatory effect, which is through the concentration-dependent inhibition of the contraction. The contraction is that isolated porcine coronary artery rings induced by KCl, histamine, 5-hydroxytryptamine, and extracellular Ca^2+^ (Zhang et al., 2009).

In clinical settings, the combination of BCS with conventional drugs, such as antiplatelet medications, has been shown to effectively reduce intracranial pressure and improve circulation for CI. This combined approach yields significant therapeutic benefits, improving neurological deficit scores, blood lipid profiles, and hemorheological parameters (Cai et al., 2004).

Overall, the above findings indicate that BCS and Tau have the effects of alleviating microcirculation disturbance after CI and promoting complete reperfusion. The therapeutical effects are achieved by inhibiting thrombosis and dilating blood vessels during the acute stage injury of CI.

#### 5.1.2 Suppressing neuronal excitotoxicity

Glutamate and aspartate are examples of excitatory amino acids (EAA), while gamma-aminobutyric acid (GABA) and glycine (Gly) are examples of inhibitory amino acids (IAA). All of these are neurotransmitters in the central nervous system. Following the occurrence of CI, neurons release large amounts of EAA, such as glutamate and aspartate. Due to insufficient ATP supply and diminished EAA reuptake, the accumulation of EAA triggers excitatory neurotoxicity by stimulating their respective receptors. This cascade of events is linked to inflammatory responses, oxidative stress, leading to neuronal demise (Deng et al., 2004; Korde et al., 2007; Xu et al., 2007; Li X. X. et al., 2016). Therefore, it is imperative to counteract the detrimental effects of amino acid overstimulation to alleviate the immediate damage caused by CI.

Tau serves as a prevalent inhibitory neurotransmitter in the central nervous system (Huxtable, 1992). Studies indicate that Tau in NBC comprises approximately 4.71% of the total amino acid content (Wen et al., 2020). Noticeably, Tau exhibits an anti-EAA toxicity effect (Wu and Prentice, 2010) by reducing neuronal excitability through the activation of γ-aminobutyric acid type A receptors (GABA_A_R), increasing Cl^−^ influx, and hyperpolarizing the cell membrane (Wang G. H. et al., 2007). Additionally, Tau can mitigate hypoxia induced by focal CI, or the influx of Ca^2+^ caused by N-methyl-D-aspartate (NMDA). By modulating the transmembrane movement of Ca^2+^, stabilizing the intracellular Ca^2+^ concentration, enhancing the energy barrier, and inhibiting the release of glutamate and aspartic acid (Asp), Tau aids in reducing EAA neurotoxicity (Sun et al., 2008; Zhao et al., 2012). Furthermore, TUDCA has been demonstrated to lower serum glutamate levels and inhibit EAA toxicity in rats subjected to transient CI in animal studies (Bian et al., 2019). Other studies demonstrated that NBC could elevate the levels of IAA, such as GABA and Gly in the rat brain striatum, thereby exert anti-EAA toxicity (Liu P. et al., 2010).

**FIGURE 2:**
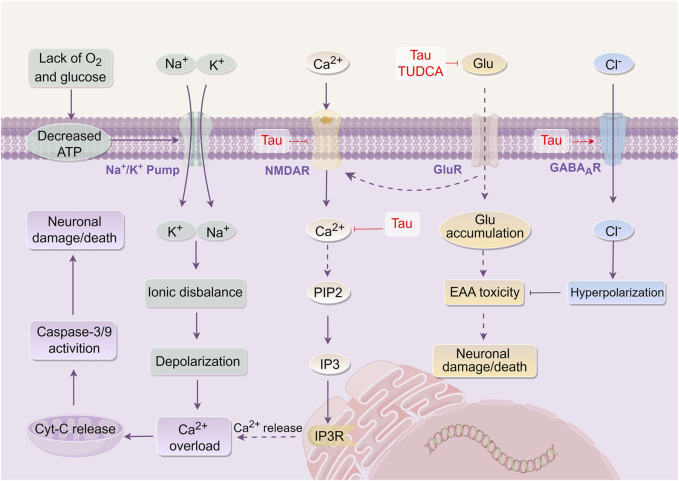
Mechanism of *Bovis Calculus* against excitatory amino acid toxicity in CI. Energy disturbances in mitochondria, abnormal ion pump function, and cell depolarization. Glutamate and other EAA are released into the intercellular space. Glutamate activates GluR, such as NMDAR, leading to the promotion of Ca^2+^ influx. This influx then produces IP3-3K, activating the binding of IP3 to endoplasmic reticulum IP3R, resulting in endoplasmic reticulum stress and the release of stored Ca^2+^ from the endoplasmic reticulum. The accumulation of Ca^2+^ in cells inhibits the function of mitochondria and worsens the disruption of energy metabolism. 1). Tau promotes the influx of Cl^−^ and hyperpolarizes the cell membrane by activating GABA_A_R and opening the chloride channel. 2). Tau promotes Ca^2+^ outflow and reduces intracellular Ca^2+^ concentration by inhibiting the activation of NMDAR. 3). Tau inhibits the binding of glutamate to its receptor, which ultimately affects endoplasmic reticulum stress and inhibits the release of Ca^2+^ from endoplasmic reticulum storage. 4). Tau and TUDCA can decrease the level of extracellular EAA and alleviate their toxicity. Abbreviations: ATP, Adenosine triphosphate; Tau, Taurine; TUDCA, Tauroursodeoxycholic acid; NMDAR, N-methyl-D-aspartate receptor; Glu, Glutamic acid; GABA_A_R, γ-aminobutyric acid type a receptors; CytC, Cytochrome C; PIP2, phosphatidylinositol (4,5)bisphosphate; IP3, Inositol triphosphate; EAA, Excitatory amino acids.

The aforementioned findings indicate that the active components of Tau and TUDCA exert anti-EAA toxicity by reducing EAA levels, activating GABA_A_R, and increasing IAA content, thereby eliciting protective effects on the brain (Figure 2).

#### 5.1.3 Anti-oxidative damage

CI/RI is closely associated with oxidative stress as a prominent pathological mechanism. Upon the occurrence of CI, an excess of reactive oxygen species (ROS) is generated. These ROS disrupt the delicate balance of the intracellular oxidation-antioxidation system. Besides, the excessive accumulation of ROS causes oxidative damage to lipids, proteins, and tissue cells. This oxidative stress triggers various signaling pathways, including mitochondrial and endoplasmic reticulum pathways, leading to the release of Cyt-C and apoptosis-inducing factors, ultimately resulting in nerve cell apoptosis (Wang and Huang, 2012; Rodrigo et al., 2013; Orellana-Urzúa et al., 2020). Thus, mitigating oxidative stress injury contributes to alleviating symptoms during the acute stage of CI.

BR, a key active component of bile components, serves as a potent antioxidant (Suh et al., 2018). It effectively reduces oxidative damage by enhancing the activity of antioxidant enzymes such as superoxide dismutase (SOD) and glutathione peroxide (GSH-Px). Furthermore, it diminishes the levels of oxidative injury markers, including malondialdehyde (MDA) and 8-hydroxy-2′-deoxyguanosine (8-OHdG) (Zhang et al., 2016). BV displays the ability to inhibit the generation of superoxide anion (O_2_^·-^), lower lipid peroxidation levels, and reduce DNA oxidative damage. Additionally, it decreases MDA and 8-OHdG content while downregulating the expression of inducible nitric oxide synthase (iNOS) (Deguchi et al., 2008; Li et al., 2017).

CA, contained in BC, has been shown to elevate SOD levels and reduce MDA content following oxygen-glucose deprivation/reperfusion (OGD/R) in the neurovascular unit (NVU), exerting antioxidant effects (Li C. X. et al., 2020). TUDCA is one of the essential bile acid components of BC. It has the ability to reduce serum MDA and oxidized low-density lipoprotein levels, enhance antioxidant SOD and GSH-Px levels, and modulates oxidative stress injury through the Nrf2-VLDLR pathway in rats with transient middle cerebral artery occlusion (Bian et al., 2019). HDCA, present in BC, plays a significant role in regulating oxidative stress post-OGD/R in the NVU and exhibits an antioxidative stress injury effect (Li C. X. et al., 2019).

**FIGURE 3:**
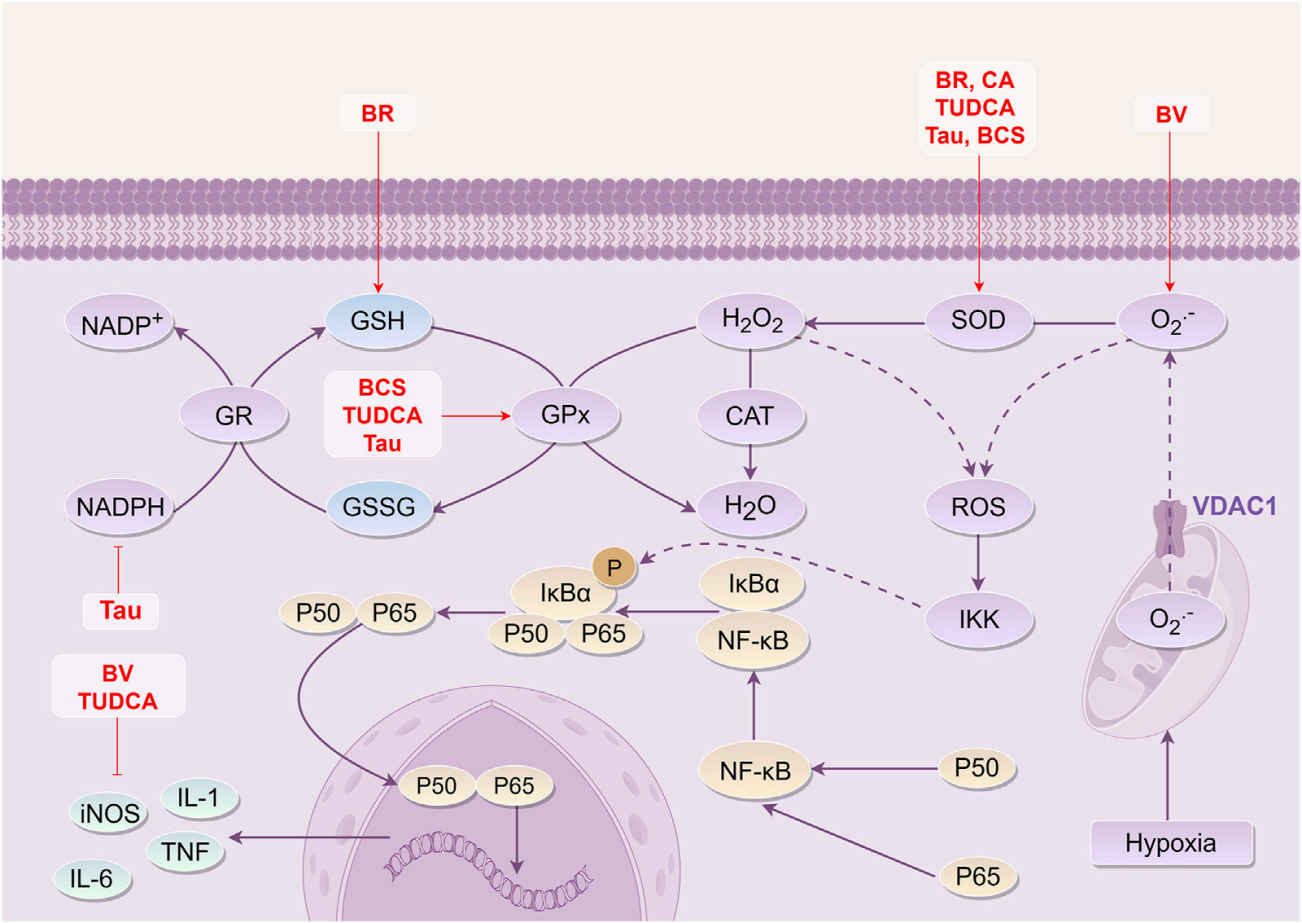
Mechanism of *Bovis Calculus* against oxidative stress injury in CI. Mitochondrial hypoxia disrupts ATP synthesis, causing a significant release of oxygen ions through the voltage-dependent anion channel 1 (VDAC1) on the mitochondrial membrane, which in turn disrupts the intracellular oxidation-antioxidation balance system. ROS reacts with intracellular organelles and phospholipid membranes, leading to cell damage. 1). BR can increase the content of SOD and GSH. 2). BV can inhibit the production of O_2_^·-^ and the expression level of iNOS. 3). CA can increase the content of SOD. 4). TUDCA can inhibit the expression of iNOS and increase the content of SOD and GSH-Px. 5). Tau can inhibit the transformation between NADPH and NADP^+^, increase the content of SOD and GSH-Px, and reduce the production of ROS. 6). BCS can increase the content of SOD and GSH-Px to play a role in anti-oxidative stress injury. Abbreviations: NADPH, Nicotinamide adenine dinucleotide phosphate; GR, gluathione reductase; GSH, Glutathione; GSSG, Glutathione (Oxidized); Gpx, Glutathione peroxidase; CAT, Catalase From *Micrococcus* Lysodeikticus; SOD, Superoxide dismutase; ROS, Reactive oxygen species; VDAC1, Voltage Dependent Anion Channel 1; IκBα, I-kappa-B-alpha; NF-κB, Nuclear factor-κb; IKK, IκB kinase; iNOS, Inducible nitric oxide synthase; IL-1, Interleukin-1; IL-6, Interleukin-6; TNF, Tumor necrosis factor; BR, Bilirubin; BCS, *Bovis Calculus sativus;* TUDCA, Tauroursodeoxycholic acid; Tau, Taurine; BV, Biliverdin; CA, Cholic acid.

Although Tau within BC does not directly scavenge O_2_^·-^, it can eliminate other oxygen radicals, such as hydroxyl radicals, thereby reducing tissue damage caused by oxygen radicals. This action enhances SOD, total anti-oxidant capacity (T-AOC), and GSH-Px levels, reduces MDA content, and inhibits the activity of nicotinamide adenine dinucleotide phosphate (NADPH) to confer antioxidant effects (Raschke et al., 1995; Hanna et al., 2004; Sun et al., 2008; Fang and Peng, 2013; Han et al., 2016; Zhu et al., 2016; Cai et al., 2018). Furthermore, BCS has been shown to enhance cellular tolerance to hypoxia, suppress free radical generation, and modulate neurotransmitters. These effects are linked to the augmentation of SOD and GSH-Px activity as well as T-AOC by BCS (Cai et al., 2007). Collectively, the findings suggest that BR, BV, TUDCA, HDCA, and Tau, as the active components of BCS or NBC, have antioxidant effects against stress injuries. These mechanisms involve scavenging oxygen free radicals or enhancing antioxidant activity (Figure 3).

### 5.2 Mechanisms of against CI/RI in the subacute stage

During the subacute stage, alterations in BBB permeability and the release of signaling molecules,such as cytokines, by astrocytes, microglia, and oligodendrocytes contribute to inflammatory injuries. In turn, reperfusion initiates a cascade of free radicals that result in apoptosis by inducing mitochondrial damage, cytochrome-C (Cyt-C) release, and activation of multiple pathways. These interconnected factors create a “waterfall” cascade reaction of IS, ultimately leading to various forms of neuronal cell death, including necrosis and apoptosis, through continuous accumulation (Campbell et al., 2019).

#### 5.2.1 Alleviating inflammation response

Excessive local inflammatory response tends to worsen brain damage during CI, leading to a poor prognosis (Lawrence, 2009; Cheng et al., 2020). Following CI, the build-up of ROS triggers the activation of complement, platelets, and endothelial cells, resulting in inflammatory substances generation such as interleukin-6 (IL-6), interleukin-1β (IL-1β), and tumor necrosis factor-α (TNF-α). Moreover, necrotic neurons release nucleosides that stimulate purine receptors on microglia and circulating macrophages. The process leads to the aggregation of inflammatory cells and the infiltration of neutrophils (Iadecola and Anrather, 2011; Fluri et al., 2015). According to research, Nuclear factor-κB (NF-κB) is activated by the phosphorylation of inflammatory transcription factors during inflammation. This activation allows NF-κB to enter the nucleus and regulate cytokine expression, leading to the production of numerous adhesion molecules. Simultaneously, inflammatory factors and adhesion molecules further induce NF-κB phosphorylation, intensifying the inflammatory response (Sun et al., 2014; Zhao H. et al., 2018). Therefore, anti-inflammatory measures are crucial for alleviating adverse symptoms following CI-reperfusion.

Different researches have revealed that CA, UDCA, TUDCA, HDCA, and Tau present in BC, possess anti-inflammatory properties that can reduce levels of TNF-α, IL-6, and IL-1β in CI. Additionally, these compounds demonstrate the capacity to inhibit the activation of NF-κB and mitigate the inflammatory response (Joo et al., 2003; Zhu et al., 2004; Hua et al., 2009; Wang and Xu, 2017; Bian et al., 2019; Li C. X. et al., 2020).

Among these constituents, CA has been shown to suppress TNF-α and IL-1β expression post-CI, reducing monocyte chemoattractant protein-1 (MCP-1) expression at the site of CI. Moreover, CA has the ability to impede microglia activation and the aggregation of monocyte macrophages to the injury site (Zhu et al., 2004; Hua et al., 2009; Li C. X. et al., 2020). Furthermore, UDCA has shown the ability to lower NO levels, decrease IL-1β and TNF-α expression, and inhibit microglia activation (Joo et al., 2003).

Specifically, TUDCA has the ability to stimulate adenylate cyclase and enhance cyclic adenosine monophosphate (cAMP) expression. TUDCA activates Takeda G-protein receptor 5 (TGR5) in microglia and triggers cyclic adenosine-dependent protein kinase A and cyclic adenosine-independent protein kinase A pathways to regulate the transcription of pro- and anti-inflammatory proteins. TUDCA also reduces the levels of high-sensitivity c-reactive protein (hs-CRP), TNF-α, and interleukin-8 (IL-8) through transcription and translation inhibition. Additionally, it negatively regulates the Nrf2 inflammatory pathway, contributing to its anti-inflammatory effects (Yanguas-Casás et al., 2017; Bian et al., 2019). Additionally, HDCA significantly reduces inflammatory response in NVU following OGD/R, and may exert anti-inflammatory effects through its conversion to bile acids like CA and TUDCA (Li C. X. et al., 2019).

Furthermore, Tau acts as a suppressive amino acid in the brain, suppressing NF-κB activation, and preventing neutrophil infiltration and neuron apoptosis by hindering microglia activation in ischemic tissues. Tau also hinders NO production, suppresses pro-inflammatory cytokines such as TNF-α, IL-1β, and IL-6, as well as adhesion molecules like intercellular adhesion molecule-1 (ICAM-1) and vascular cell adhesion molecule-1 (VCAM-1), and prostaglandin E2 (PGE2) (Luo et al., 2004; Yang et al., 2006; Sun et al., 2011; Wang and Xu, 2017; Wang et al., 2018). BV, another essential bile pigment in BC, exerts anti-inflammatory effects by down-regulating inflammatory mediators expression, including TNF-α, IL-6, IL-1β, and iNOS mRNA, and negatively regulating heme oxygenase-1 mRNA expression (Li et al., 2017; 2021).

Clinical studies have indicated that BCS is effective in reducing the inflammatory response in patients with CI, thereby decreasing neurological damage and providing neuroprotective effects (Wang et al., 2021).

**FIGURE 4:**
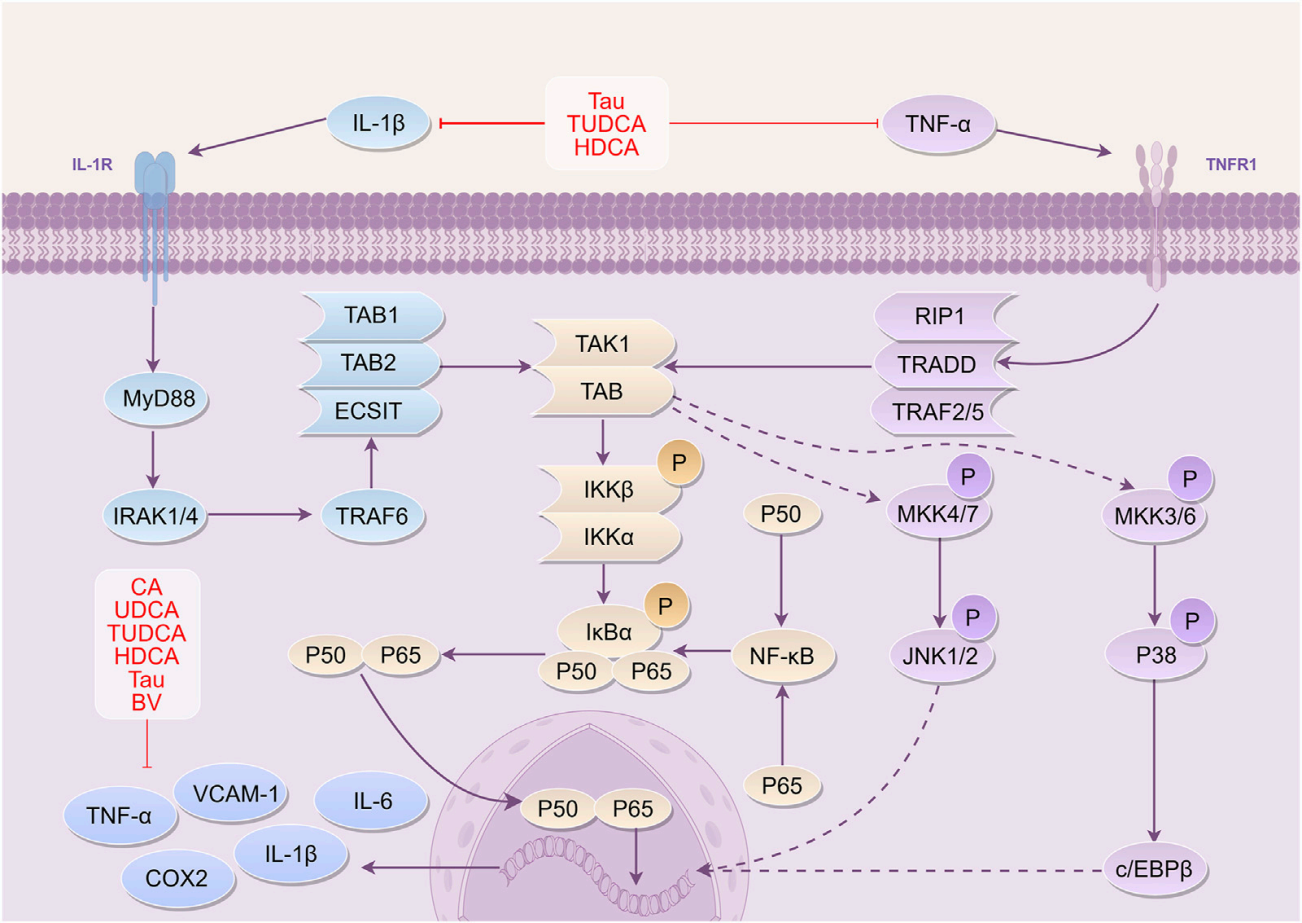
Mechanism of *Bovis Calculus* against inflammation in CI IL-1β binds to its receptor IL-1R and ultimately activates IκBα phosphorylation. NF-κB enters the nucleus to participate in the transcriptional expression of DNA and produce inflammatory cytokines. Binding of TNF-α to its receptor TNFR1 similarly phosphorylates and activates IκBα, allowing NF-κB to enter the nucleus. 1). CA inhibits the expression of inflammatory factors and adhesion molecules, reducing the accumulation of monocytes at the site of injury. 2). UDCA inhibits the expression of IL-1β and TNF-α and suppresses the activation of microglia. 3). TUDCA reduces the production of inflammatory factors and inhibits their binding to receptors. 4). Tau reduces TNF-α and IL-1β levels and inhibits their binding to receptors. 5). BV can downregulate the expression of inflammatory mediators such as TNF-α, IL-6, and IL-1β, thereby reducing inflammatory damage. 6). HDCA can inhibit the expression of inflammatory factors and can be converted into components such as CA and TUDCA, which exert neuroprotective effects. Abbreviations: IL-1β, Interleukin-1β; TNF-α, Tumor necrosis factor-α; TNFR1, tumor necrosis factor receptor 1; IRAK1, IL-1 receptor associated kinase 1; IRAK4, IL-1 receptor associated kinase 4; TRAF6, Tumor necrosis factor receptor-associated factor 6; ECSIT, Evolutionarily conserved signaling intermediate in Toll pathway; TAK1, TGF beta-Activated Kinase 1; TAB1, TGF-beta-activated kinase 1-binding protein 1; IKKβ, inhibitor of kappa B kinase β; IKKα, inhibitor of kappa B kinase α; IκBα, I-kappa-B-alpha; NF-κB, Nuclear factor-κb; RIP1, receptor-interacting protein 1; TRADD, tumor necrosis factor receptor-associated death domain protein; TRAF2, tumor necrosis factor receptor-associated factors 2; TRAF5, tumor necrosis factor receptor-associated factors 5; MKK4, Mitogen-activated protein kinase kinase 4; MKK7, Mitogen-activated protein kinase kinase 7; JNK1, c-Jun N-terminal kinase 1; JNK2, c-Jun N-terminal kinase 2; c/EBPβ, CCAAT/enhancer binding protein beta; TNF-α, Tumor necrosis factor-α; VCAM-1, Vascular cell adhesion molecule-1; IL-6, Interleukin-6; COX2, Cyclooxygenase-2; TUDCA, Tauroursodeoxycholic acid; Tau, Taurine; BV, Biliverdin; CA, Cholic acid; UDCA, Ursodeoxycholic acid.

In summary, the active constituents of BC, CA, UDCA, TUDCA, HDCA, and Tau exhibit anti-inflammatory effects. They achieve that by suppressing the production of inflammatory factors IL-1β, IL-6, and TNF-α. Additionally, these active constituents reduce the expression of MCP-1 at ischemic sites, lower NO levels, increase cAMP and activate TGR5. Furthermore, they regulate the transcription of pro- and anti-inflammatory proteins, reduce hs-CRP, inhibit microglia activation, suppress ICAM-1, VCAM-1, and PGE2, decrease iNOS mRNA, and regulate heme oxygenase-1 mRNA expression (Figure 4).

#### 5.2.2 Protecting the blood-brain barrier

The BBB is a complex structure consisting of cerebral microvascular endothelial cells, endothelial cell occluding, and claudin, intercellular zonula occludens-1 (ZO-1), astrocyte terminal foot, pericyte, and basement membrane (Mark and Davis, 2002; Lampugnani and Dejana, 2007). More importantly, the BBB is responsible for maintaining the overall stability of the central nervous system and safeguard the brain from the infiltration of detrimental substances.

When CI occurs, the BBB function is compromised, leading to increased permeability. This allows harmful substances and cytokines to enter the brain to penetrate the brain tissue, causing damage (Marín-Teva et al., 2004; Choi et al., 2008; 2008; Hjort et al., 2008). Noticeably, when large amounts of matrix metalloproteinases are released, the tight junctions of the BBB are impaired, and permeability is increased (Vecsernyés et al., 2014). Additionally, increased expression of water channel proteins like aquaporin-4 (AQP-4) can result in hemorrhagic brain edema (Zhang H. et al., 2020; Shi et al., 2021). Additionally, endothelial cells express amounts of inflammatory substances such as TNF-α, IL-6, IL-1β, and ICAM. These substances enhance the permeability of vascular endothelial cells and contribute to the accumulation, adhesion, and infiltration of leukocytes in the area affected by infarction. Furthermore, ICAM-1-mediated neutrophil adhesion after reperfusion blocks microcirculatory channels and exacerbates tissue damage in CI (Amantea et al., 2007; Tuttolomondo et al., 2008; Vakili et al., 2011; Treda et al., 2016). Therefore, protecting the BBB’s integrity is crucial in enhancing the therapeutic effect of cerebral ischemia-reperfusion and alleviate patients’ symptoms.

Tau has been shown to reduce secondary brain injury after CI by down-regulating the expression of matrix metalloproteinases-3 (MMP-3) in brain-injured rats, thereby protecting the BBB (Li et al., 2010). Moreover, GUDCA inhibits the activation of MMP-9 and caspase-9 in a model of superoxide dismutase-1-induced neuronal degeneration, thereby exerting a protective effect on the BBB (Vaz et al., 2015). Additionally, the CA can protect the BBB function following NVU OGD/R. The mechanism is associated with the reduction of serum factor permeability coefficient and the increase in transepithelial electrical resistance (TEER) value and Gamma-glutamyl transferase activity (γ-GT) (Li C. X. et al., 2020).

Furthermore, investigation revealed that BC and its active constituents, including Tau, TUDCA, and UDCA, can decrease lactate dehydrogenase levels, increase γ-GT content, raise TEER values, and decrease fluorescein sodium permeability coefficient. These medications alone can lower the expression levels of inflammatory factors TNF-α, IL-6, and IL-1β, and reduce the protein expression of MMP-2 and MMP-9. Among them, Tau alone can stimulate the protein expression of tight junction protein ZO-1, occludin, and claudin-5 (Zhang et al., 2019). Combining Tau with UDCA can inhibit the MyD88/NF-κB pathway, while Tau and TUDCA combination can suppress the P38MAPK pathway, thereby exerting cerebral protective effects (Xu et al., 2017).

In conclusion, BC and its bioactive constituents, such as Tau, GUDCA, CA, TUDCA, and UDCA, collectively inhibit caspase-9 activation, reduce serum factors permeability coefficient, improve TEER and γ-GT activity, decrease inflammatory factors expression, block the MyD88/NF-κB and P38MAPK pathways, and promote the expression of tight junction proteins such as ZO-1, occludin, and claudin-5. These mechanisms play a crucial role in protecting the BBB’s integrity.

#### 5.2.3 Anti-apoptosis

Apoptosis plays a crucial role in neuronal death following CI. After CI, the brain has two important regions that are independent of ischemia: the ischemic core and the ischemic penumbra (Kulik et al., 2008). Insufficient ATP supply renders Ca^2+^-related transporter proteins ineffective. Conversely, when EAA is released and binds to EAA receptors on the postsynaptic membrane, it induces cell depolarization. This leads to a significant influx of Ca^2+^, triggering the activation of Ca^2+^ proteases, the hydrolysis of cellular proteins, and ultimately resulting in neuronal death (Radak et al., 2017; Sekerdag et al., 2018).

The accumulation of calcium ions within cells can potentially induce the release of Cytochrome C (CytC) in mitochondria, which activates caspase-9 and the final executor protein caspase-3. This process leads to the breakdown of functional proteins within cells, causing DNA damage, and ultimately initiating apoptosis (He et al., 2020; Tuo et al., 2022). In addition, cell injury induces endoplasmic reticulum stress, which is an important site for intracellular protein translation, synthesis, modification, folding, and storage of Ca^2+^. This stress will lead to the accumulation of unfolded proteins, disrupting Ca^2+^ homeostasis, and ultimately resulting in a further increase in intracellular Ca^2+^ concentration (Xu et al., 2017). Thus, the mitigation of symptoms during the subacute phase of CI and the enhancement of recovery outcomes are significantly influenced by preventing neuronal cell death.

Additionally, CA has been shown to inhibit the expression of caspase-9, caspase-3, and Bax after NVU OGD/R while increasing the expression of Bcl-2, exerting an anti-apoptotic effect (Li C. X. et al., 2020). Different researches have shown that Tau effectively prevents cell death and tissue damage by suppressing Bax activity, thus preventing the release of Cyt-C from mitochondria to the cytoplasm. Tau safeguards mitochondrial function, inhibits caspase-3 and calpain activity, and enhances the production of the anti-apoptotic protein Bcl-2. These actions help regulate the Bax/Bcl-2 ratio, ultimately resulting in anti-apoptotic effects (Schaffer et al., 2002; Zhang, 2006; Sun et al., 2011; Zhu et al., 2016).

Furthermore, Tau has been found to suppress endoplasmic reticulum stress by inhibiting the activation of transcriptional activator 6 and inositol-requiring enzyme-1 molecular pathways (Gharibani et al., 2013). TUDCA, as an important component of BC, inhibits the phosphorylation activation of protein kinase R-like endoplasmic reticulum kinase, eukaryotic initiation factor 2α, and transcriptional activator 4 expression in cortical and hippocampal regions. TUDCA also blocks caspase-12-dependent endoplasmic reticulum stress-mediated apoptosis (Rodrigues et al., 2002; Xu et al., 2017; Chen et al., 2020).

The active constituents of BC, BR, and BV, play a crucial role in inhibiting apoptosis. This is achieved by reducing the levels of caspase-3, Bax, and other proteins associated with apoptosis, while simultaneously elevating the concentration of Bcl-2 (Zhang et al., 2016; Li et al., 2021). A study demonstrated that pretreatment with BCS reduced brain swelling, damaged tissue size, neuronal cell death, and neurological impairments in rats. BCS also decreased the number of dying neurons, possibly by suppressing caspase-9 and caspase-3 activity, and Cyt-C release. This inhibition of mitochondria-mediated apoptotic signaling contributed to CI/RI reduction (Lu et al., 2020).

**FIGURE 5:**
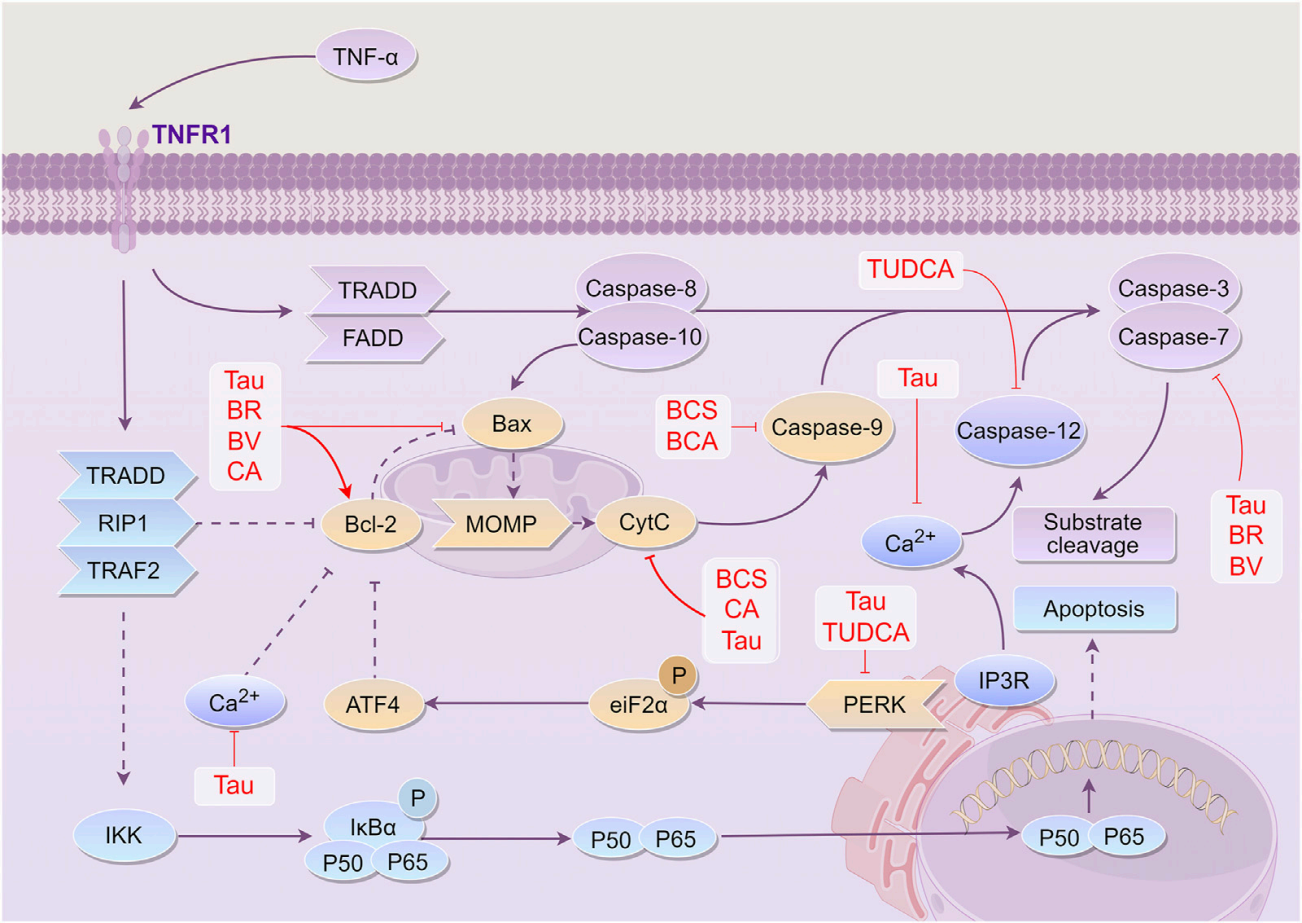
Mechanism of *Bovis Calculus* against cell apoptosis in CI. The ratio of Bcl-2 to Bax, a group of proteins involved in apoptosis and anti-apoptosis, determines whether cells are more likely to survive or die. TNF-α promotes the expression of Bax and inhibits the expression of Bcl-2 by activating the TNFR1 receptor. This activation eventually leads to the production of the apoptotic end protein caspase-3, which breaks down most of the functional proteins in the cell, resulting in apoptosis. 1). Tau inhibits the expression of caspase-3, the release of Ca^2+^, activation of the PERK pathway, the expression of Bax, release of Cyt-C, and expression of caspase-9, and promotes the expression of Bcl-2. 2). BV inhibits the expression of caspase-3 and Bax and promotes the expression of Bcl-2. 3). BR promotes the expression of Bcl-2 and inhibits the expression of Bax and caspase-3. 4). CA can inhibit the expression of Bax, the release of Cyt-C, and the expression of caspase-9, and promote the expression of Bcl-2. 5). BCS can inhibit the release of Cyt-C and the expression of caspase-9. 6). TUDCA can inhibit the expression of the PERK pathway and CASP12 activated by endoplasmic reticulum stress, thus playing a role in cell protection and preventing apoptosis. Abbreviations: TNF-α, Tumor necrosis factor-α; TNFR1, tumor necrosis factor receptor 1; TRADD, tumor necrosis factor receptor-associated death domain protein; FADD, Fas-associating protein with a novel death domain; TRAF2, tumor necrosis factor receptor-associated factors 2; Bcl-2, B-cell lymphoma-2; Bax, Bcl-2 associated X protein; MOMP, mitochondrial outer membrane permeabilization; CytC, Cytochrome C; IP3, Inositol triphosphate; ATF4, Activating transcription factor 4; eiF2α, Eukaryotic translation initiation factor 2α; PERK, protein kinase RNA-like endoplasmic reticulum kinase; IKK, Inhibitor of kappa B kinase; IκBα, I-kappa-B-alpha; Tau, Taurine; BV, Biliverdin; CA, Cholic acid; TUDCA, Tauroursodeoxycholic acid; BR, Bilirubin; BCS, *Bovis Calculus sativus;* BCA, *Bovis Calculus artifactus*

Overall, these findings highlight that the active components of BC, CA, Tau, TUDCA, BV, and BR, can protect mitochondria, inhibit endoplasmic reticulum stress, impede apoptosis, and exert neuroprotective effects through various mechanisms of action (Figure 5).

### 5.3 Mechanisms of against CI/RI in the restoration stage

In the late phase of CI, cell proliferation and differentiation lead to predominant repair mechanisms, which are characterized by extensive reconstruction of cell populations, angiogenesis, and regeneration of myelin. Among them, neovascularization helps to relieve local microcirculation disturbance after CI and reduces CI/RI. Specifically, neurogenesis plays a reparative role by producing neurotrophic factors at the injury site, thereby promoting neuronal regeneration (Steliga et al., 2020).

#### 5.3.1 Promoting angiogenesis

The process of angiogenesis plays a crucial role in restoring blood oxygen supply to injured brain tissue and mitigating damage from IS. Additionally, within the central nervous system, there exists a significant interplay between blood vessels and axons. Angiogenesis also plays a crucial role in the growth of axons and the generation of new neurons, ultimately facilitating the development and differentiation of neurons. These contribute to restoring typical brain function (Millikan, 1970; Hatakeyama et al., 2020). Hence, the promotion of angiogenesis is crucial for brain function repair during the recovery phase following an IS.

Vascular endothelial growth factor (VEGF) has mitogenic and anti-apoptotic effects on endothelial cells and plays a pivotal role in angiogenesis and remodeling in the restoration stage of IS (Melincovici et al., 2018). According to research, Hypoxia-inducible factor (HIF-1α) plays a key role in promoting angiogenesis after IS by regulating the expression of downstream genes (Shi, 2009). Moreover, Angiotensin-1 (Ang-1) has been found to promote cerebral angiogenesis after IS through the Mas/eNOS-dependent pathway, reduce ischemic tissue injury, and enhance its tolerance to subsequent ischemia (Jiang et al., 2014).

Studies demonstrated that Tau presence in ischemic brain tissue enhances the production of HIF-1α mRNA, thereby promoting angiogenesis, microcirculation reconstruction, and reducing ischemic brain tissue damage following IS (Wang H. R. et al., 2007). Additionally, BCS activates 3-carboxyphosphatidylinositol kinase (p-PI3K) and serine/threonine protein kinase (p-Akt) proteins, leading to an upregulation in the expression of p-PI3K, p-Akt protein, and PI3K mRNA. This activation results in increased levels of serum VEGF and Ang-1 (Ren et al., 2023). Furthermore, BCS modulates the HIF-1α/VEGF and PI3K/AKT pathways, thus aiding in the restoration of normal brain function post-IS (Zhang et al., 2019).

In conclusion, BCS and its active component Tau promote angiogenesis following IS by modulating HIF-1α/VEGF and PI3K/Akt pathways, thereby contributing significantly to the restoration of normal brain function subsequent to an IS.

#### 5.3.2 Facilitating nerve regeneration

CI can lead to limited endogenous neurogenesis within the body, failing to adequately repair nerve damage following ischemia. The rehabilitation and reconstruction of neurological function play a vital role in the treatment of IS. Studies have indicated that stem cell-based therapy, which includes cell transplantation and stimulating endogenous neurogenesis, holds promise as a potential strategy for repairing and regenerating damaged brains. This innovative approach may offer a secondary treatment window for IS patients (Zhu et al., 2018).

Tau, a natural amino acid found in the brain, has been demonstrated to exert positive effects on brain growth and development (Huxtable, 1992; Tochitani, 2017). In cases of CI/RI, Tau has been shown to enhance the expression of neural stem cell factor (SCF) mRNA in the cortex, striatum, and paraventricular zone. Moreover, it promotes nestin expression and augments the population of neural stem cells during the ischemic recovery process (Zhang et al., 2008; Zang et al., 2011).

Tau facilitates the proliferation and differentiation of hippocampal neural stem cells induced by glutamic acid (Glu). This was achieved by activating the brain-derived neurotrophic factor (BDNF)/ERK/CREB signaling pathway and repairing brain injury after CI (Zhou, 2019). BDNF plays a crucial role in neuronal growth and development. Upon binding to the tyrosine kinase B receptor (TrkB), BDNF triggers downstream signaling pathways such as mitogen-activated protein kinase (MAPK)/extracellular signal-regulated kinase (Erk) and PI3K/Akt, governing neuronal proliferation and differentiation (Choo et al., 2017; Li C. X. et al., 2020).

As one of the important free bile acids present in BC, HDCA can effectively protect NVU in NVU ODG/R. HDCA might achieve this by promoting the expression and release of BDNF and glia cell line-derived neurotrophic factor (GDNF), thereby fostering neural recovery (Li C. X. et al., 2019). Moreover, Nerve growth factor (NGF) contributes to nerve growth. After an IS, the content of NGF in brain tissue decreases, weakening the body’s protective effect. One study demonstrated that BCA can increase the content of NGF and aid in nerve recovery in the recovery stage of IS (Li and Li, 2004).

In conclusion, BC and its active constituents have a significant impact on restoring nerve function after IS. This is achieved by stimulating SCF mRNA production, activating the BDNF/ErK/CREB signaling pathway, boosting BDNF and GDNF expression, and facilitating neuronal repair.

## 6 Clinical applications

### 6.1 Clinical actions

In *Chinese Pharmacopoeia*, the actions of NBC is to clear the heart, sweep phlegm, open the orifices, cool the liver, extinguish wind, and remove toxin. In addition, the actions of BCA is to clear the heart, remove toxin, resolve phlegm and relieve fright. Furthermore, the actions of BCS is to clear the heart, sweep phlegm, open the orifices, cool the liver, extinguish wind, and remove toxin (Chinese Pharmacopoeia Committee of People’s Republic of China, 2020). Therefore, the actions of the three is similar, and NBC and BCS have the same effect. But because NBC is very expensive, its substitute BCS is widely used.

### 6.2 Clinical effects of BC

**FIGURE 6:**
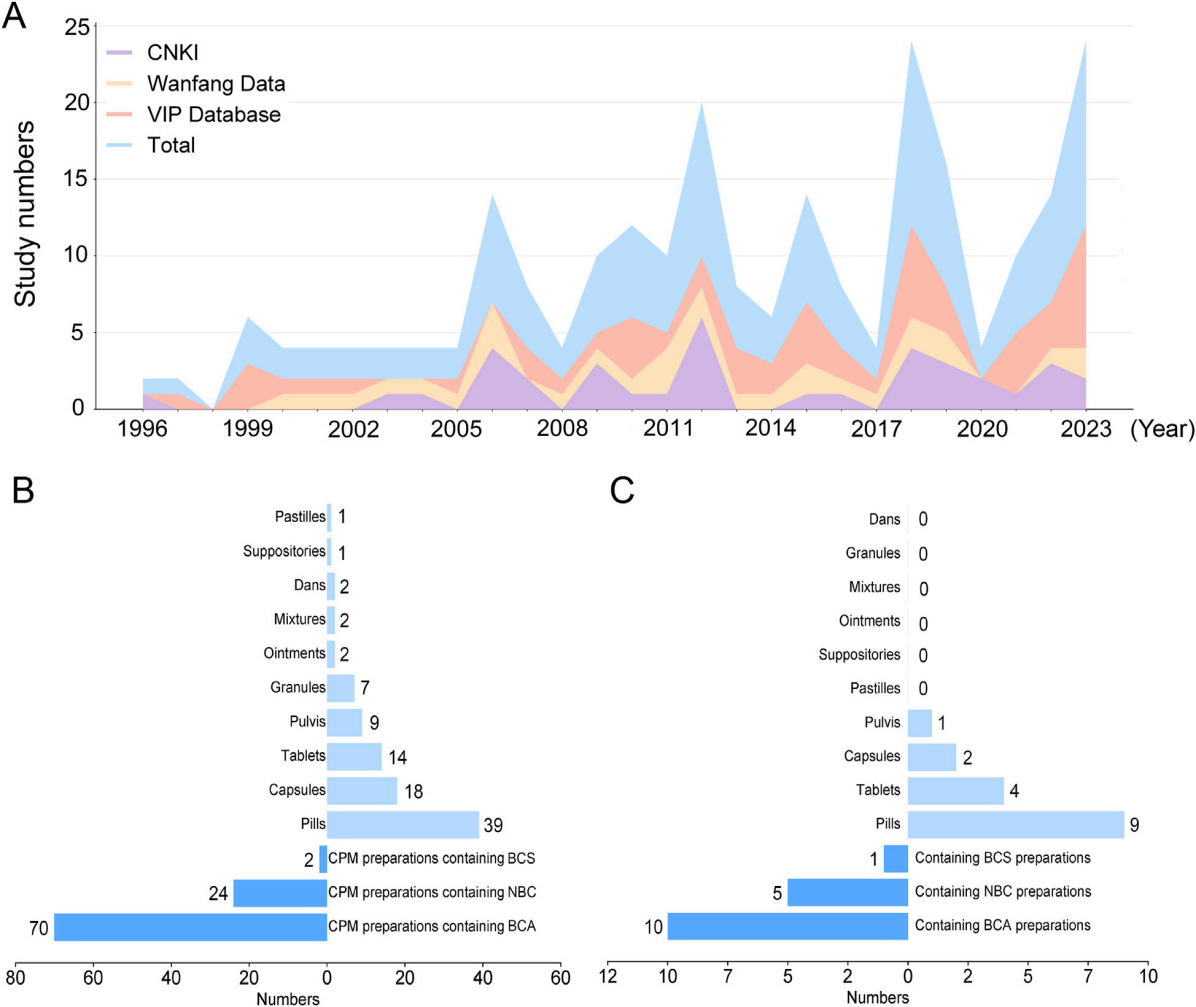
**(A)** A stacked area chart of the annual publication count and cumulative publication count for three databases each year (From 1996 to 2023) **(B)** The respective proportions of three types BC preparations in 95 preparations and the proportions of different dosage forms **(C)** The respective proportions of three types BC preparations in 16 preparations for the IS treatment and the proportions of different dosage forms Abbreviations: BCS, *Bovis Calculus sativus;* BCA, *Bovis Calculus artifactus;* NBC, Natural *Bovis Calculus.*

Currently, an increasing number of researchers are focusing on the clinical and preclinical studies of BC for IS (Figure 6A). Notably, there are 95 formulations of CPMs containing BC listed in the *Chinese Pharmacopoeia*. Among these, 16 formulations are explicitly indicated for the treatment of IS, accounting for 16.8% of the total. Therefore, from a formulations perspective, it is evident that BC is frequently used in the treatment of IS. In addition, in clinical practice, BC is also being used in combination with other drugs for IS treatment. Compared to the conventional treatment group, BC (particularly BCS and NBC) has been shown to enhance the therapeutic effects of conventional treatment. Furthermore, it has demonstrated the ability to promote the recovery of patients’ neurological function and consciousness, improve patients’ hemorheological indexes and inflammatory levels, as well as enhance the medium-to-long-term prognosis of patients (Table 3).

**TABLE 3:** Clinical application of BC in IS.

Study	Drug	Randomized controlled trial	Experimental subject		Treatment method		Course of treatment	Result	Clinical safety
			Treatment group	Control group	Treatment group	Control group			
Cai et al. (2004)	BCS	Yes	95 cases	32 cases	BCS (Three times a day, two tablets per time, 50 mg per tablet, oral application or nasal feeding)	NBC (Three times a day, two tablets per time, 50 mg per tablet, oral application or nasal feeding)	5 days	The effective rate was 86.3% in the treatment group and 80.6% in the control group	Routine blood test, urine test, stool test, liver function, and kidney function all showed no obvious abnormalities
Chen (2022)	BCS and Zhongfeng Xingnao liquid	Yes	23 cases	25 cases	BCS (Twice a day, 0.15 g per time, oral application) + Zhongfeng Xingnao liquid + Conventional treatment	Zhongfeng Xingnao liquid + Conventional treatment	14 days	1) The effective rate was 91.3% in the treatment group and 76.00% in the control group 2) The treatment group showed some improvement in NIHSS score, FOUR score, CRS-R score, and GCS score	1) The medications in the treatment regimen have no significant impact on liver function (Alanine Transaminase, Aspartate Aminotransferase), kidney function (serum creatinine), and electrolytes (K+, Na+, Ca2+); 2) The medications in the treatment regimen have no significant impact on coagulation function (APTT, INR); 3) The treatment regimen has no significant impact on the blood routine (RBC, WBC, PLT); 4) The adverse reactions related to the experimental drug were mild diarrhea, with 5 cases in the treatment group and 4 cases in the control group. Symptoms improved after taking the medication; 5) No signs of active gastrointestinal bleeding were observed
Liao (2020)	BCS and Gastrodin Injection	Yes	32 cases	32 cases	BCS (Twice a day, 0.15 g per time, nasal feeding) + Gastrodin Injection + Conventional treatment	Conventional treatment	14 days	1) The effective rate was 96.87% in the treatment group and 93.75% in the control group 2) Treatment group patients showed better improvement in ESR, HCT, TG, and LDL-C levels compared to the control group	The incidence of adverse reactions was 6.25% in the treatment group and 15.63% in the control group, this indicates that the treatment group can reduce adverse reactions
Liu et al. (2010b)	BCS	Yes	45 cases	45 cases	BCS (Twice a day, oral application or nasal feeding) + Conventional treatment	Conventional treatment	14 days	1) The effective rate was 95.6% in the treatment group and 84.4% in the control group 2) The treatment group showed a significant decrease in WBV, HCT, and FIB levels 3) The treatment group showed a significant decrease in cholesterol, TG, and LDL-C levels	There were no obvious adverse reactions in all cases, no significant changes in liver, kidney function and blood routine, and no bleeding tendency
Peng et al. (2019)	BCS	Yes	40 cases	40 cases	BCS (Twice a day, 0.3 g per time) + Conventional treatment	Vinpocetine Injection + Conventional treatment	14 days	1) The effective rate was 95.00% in the treatment group and 80.00% in the control group 2) The NIHSS score of the treatment group was significantly lower than that of the control group	The incidence of adverse reactions was 2.50% in the treatment group and 17.50% in the control group
Wang et al. (2021)	BCS	Yes	54 cases	54 cases	BCS (0.3 g per day, nasal feeding) + Conventional treatment	Conventional treatment	7 days	1) The GCS score of the treatment group was higher than that of the control group 2) The NIHSS score and mRS score in treatment group were lower than that of the control group 3) Serum Hs-CRP, NSE and S-100β protein levels in the treatment group was lower than that in the control group	There were no liver and kidney function damage and abnormal coagulation function
Wu et al. (2023)	BCS	Yes	49 cases	49 cases	BCS (0.3 g per day, oral application) + rt-PA thrombolytic treatment	rt-PA thrombolytic treatment	7 days	1) The treatment group has a higher GCS score than the control group 2) The mRS score in treatment group were higher than that of the control group, BCS can significantly improve the prognosis of stroke patients	_
Xiang and Xiong (2023)	BCS	Yes	50 cases	50 cases	BCS (Twice a day, 0.15 g per time) + Conventional treatment	Conventional treatment	30 days	1) The treatment group has lower ESR, HCT, TG and LDL-C levels than the control group 2) The treatment group showed a significant decrease in NSE level and NIHSS score	The treatment group had two cases of adverse reactions, while the control group had nine cases
Zeng (2024)	BCS and Huatan Tongluo Decoction	Yes	30 cases	30 cases	BCS (Twice a day, 0.15 g per time, nasal feeding or oral application) + Huatan Tongluo Decoction + Conventional treatment	BCS (Twice a day, 0.15 g per time, nasal feeding or oral application) + Conventional treatment	14 days	1) The effective rate was 93.3% in the treatment group and 76.7% in the control group 2) The NIHSS score of the treatment group was significantly lower than that of the control group 3) The TCMSSS of the treatment group was significantly better than that of the control group	_
Zhang et al. (2015)	BCS	Yes	40 cases	38 cases	BCS (Once a day, 0.15 g per time) + Conventional treatment	Conventional treatment	14 days	The average G-Pcs scores in the treatment group at each time point after treatment were higher than those in the control group	_

Abbreviations: NIHSS, national institute of healthstroke scale; FOUR, Full Outline of UnResponsiveness; CRS-R, ComaRecoveryScale-Revised; GCS, glasgow coma scale; mRS, modified rankin scale; ESR, erythrocyte sedimentation rate; HCT, red blood cell hematocrit; TG, triglycerides; LDL-C, low density lipoprotein cholesterin; APTT, activated partial thromboplastin time; INR, international normalized ratio; RBC, red blood count; WBC, white blood cell; PLT, blood platelet; WBV, whole blood viscosity; FIB, fibrinogen; NSE, neuron specific enolase; Hs-CRP, hypersensitive C-reactive protein; TCMSSS, traditional chinese medicine syndrome score scale; G-Pcs, Glasgow-Pittsburgh coma scale.

#### 6.2.1 Alleviating neurological function and state of consciousness

Quantitative indicators play a crucial role in evaluating the neurological function and consciousness status of stroke patients. For instance, a higher National Institute of Healthstroke scale (NIHSS) score indicates that a stroke may lead to more severe neurological dysfunction. Building upon the NIHSS, the Full Outline of UnResponsiveness (FOUR) score considers indicators such as breathing pattern, eye movements, and swallowing reflex in patients. In clinical practice, the use of BCS in the treatment of IS with impaired consciousness can significantly reduce the NIHSS score, improve the FOUR score, indicating a marked enhancement in the neurological function and consciousness status of patients. Additionally, BCS is safe and stable, with a low incidence of adverse reactions (Chen, 2022).

Furthermore, a lower Glasgow Coma Scale (GCS) score indicates more severe neurological damage. Newly clinical research found that BCS can increase the GCS score and improve the prognosis of patients with IS accompanied by impaired consciousness (Wu et al., 2023). It is noteworthy that Coma Recovery Scale-Revised (CRS-R) is used to assess patients’ cognitive function and neurological function. One study demonstrated that BCS can increase the CRS-R score, improving the cognitive function of patients with IS and impaired consciousness (Chen, 2022). Otherwise, a lower Modified Rankin Scale (mRS) score indicates a better consciousness state in patients. In a new clinical study, treatment with BCS for IS patients can lower the mRS score, leading to a favorable prognosis (Wu et al., 2023).

#### 6.2.2 Improving the hemorheological indicators levels

Clinical studies have shown that elevated levels of cholesterol, triglycerides (TG), and low-density lipoprotein cholesterol (LDL-C) are closely associated with the occurrence of stroke. For example, abnormal levels of erythrocyte sedimentation rate (ESR), hematocrit (HCT), whole blood viscosity (WBV), and fibrinogen (FIB) can lead to abnormal blood viscosity, resulting in reduced blood rheology. Otherwise, clinical trials found that BCS has been shown to significantly improve cholesterol, TG, and LDL-C levels, alleviate symptoms of acute IS, promote the recovery of neurological function, improve CBF, and enhance clinical treatment outcomes (Liao, 2020). Additionally, BCS can lower HCT, WBV, and FIB levels, improve blood viscosity, and enhance blood circulation (Liu X. Y. et al., 2010).

#### 6.2.3 Lowering inflammation levels

Serum levels of hs-CRP, neuron specific enolase (NSE), and S-100β protein are commonly used to evaluate neurological damage. Hs-CRP serves as an inflammatory marker, and elevated levels indicate the presence of an inflammatory response in patients. S-100β protein levels can reflect the severity of central nervous system inflammatory responses. The serum levels of hs-CRP and NSE are associated with the severity and prognosis of IS. Noticeably, NSE can serve as a quantitative index of central nervous system damage (Pandey et al., 2014; Selçuk et al., 2014). Clinically, BCS can reduce serum levels of hs-CRP, NSE, and S-100β protein, effectively reducing central nervous system inflammatory responses, alleviating neurological damage, improving medium-to-long-term prognosis, and possessing high clinical utility (Wang et al., 2021).

### 6.3 Combination drug therapy

The positive therapeutic impact of BC and its active constituents on IS injury has been demonstrated. When combined with other medications, BC can enhance therapeutic outcomes and minimize adverse reactions. According to research, NBC combined with *Gardenia jasminoides* have the ability to increase SOD activity and MDA content in brain tissue after 72 h of ischemia-reperfusion, reduce the injury from lipid peroxidation, and enhance brain tissue antioxidant capacity (Li et al., 2004). Additionally, CA combined with geniposide has been shown to reduce the levels of inflammatory factors such as TNF-α, IL-1β, and ICAM-1 in ischemic tissue, thereby decreasing the inflammatory response (Zhu et al., 2004). Moreover, Tau combined with *Panax notoginseng* saponins improved SOD and MDA levels in ischemic tissue and enhanced the antioxidant capacity in ischemic brain tissue (Fang and Peng, 2013). Noticeably, the simultaneous utilization of BC and gardenia has the potential to enhance NGF expression levels and facilitate nerve regeneration (Li and Li, 2004). In rats, the administration *Bovis Calculus*-Fel Uris-Moschus contribute to increase rCBF, lower mean arterial blood pressure, and dilate guanylate cyclase-related cerebral vessels (Park et al., 2013). Guanylate cyclase (GC) is an enzyme that converts guanosine triphosphate (GTP) into cyclic guanosine monophosphate (cGMP), a second messenger that plays a critical role in various physiological processes, including vasodilation. In the context of cerebral vessels, guanylate cyclase is involved in mediating the effects of nitric oxide (NO), which is produced by endothelial cells in response to various stimuli. Activation of guanylate cyclase by nitric oxide leads to an increase in cGMP levels, resulting in smooth muscle relaxation and subsequent vasodilation of cerebral blood vessels. This mechanism is crucial for regulating cerebral blood flow and maintaining adequate oxygen supply to the brain (Xiong et al., 2023) Otherwise, Gastrodia elata contains various active compounds, such as gastrodin, which have been shown to have antioxidant effects and promote neuronal survival following ischemic injury. Research indicates that these compounds can enhance cerebral blood flow and improve neurological outcomes by mitigating oxidative stress and inflammation in the brain. Thus, when combined with BCS, they may play a crucial role in improving not only the peripheral venous blood sedimentation rate, HCT, TG, and LDL-C in patients with acute CI, but also in enhancing the overall rehabilitation of neurological functions (Liao, 2020).

In the laboratory, Tau combined with diazepam effectively activate both GABA and Gly receptors, hyperpolarize the cell membrane, reduce neuronal excitability, antagonize the EAA toxicity after CI, and regulate the synthesis and release of EAA and ROS (Wang et al., 2010). In addition, Tau combined with strychnine could promote nerve repair in the CA1 area of the hippocampus of ischemic rats. Result in the density of surviving neurons was significantly higher than that in the control group (Li, 2006). One study demonstrated that the combination of Tau and tPA had the ability to reduce the risk of stroke hemorrhagic transformation and secondary thrombosis of microvessels. This is achieved through inhibiting the CD147 protein-dependent MMP-9 pathway in endothelial cells of rats with CI (Jin et al., 2018). Furthermore, Tau combined with urokinase has been shown to significantly improve the infarct volume, prolong the treatment time window, enhance the neurological prognosis, and reduce the risk of hemorrhagic transformation after IS in rats with CI (Guan et al., 2011).

### 6.4 Analysis of preparations containing BC

We analyzed the proportions of three types of BC in 95 preparations and the proportions of various dosage forms. Among these CPM preparations, BCA is the most widely used with 70 preparations (73.7%), 24 preparations (25.3%) containing NBC, and two preparations (2.1%) containing BCS. Specifically, the type of BC used in Xihuang pill is either NBC or BCS. Besides, our findings indicate that the clinical applications of BC mainly include 10 dosage forms, including 39 pills (41.1%), 18 capsules (18.9%), 14 tablets (14.7%), 9 pulvis (9.5%), 7 granules (7.4%), 2 ointments (2.1%), 2 mixtures (2.1%), 2 dans (2.1%), one suppository and one pastille. It is noteworthy that among the 39 pills, Angong Niuhuang Pill is the most widely used (Figure 6B).

Furthermore, we analyzed the proportions of three types of BC in IS treatment (16 preparations) and the proportions of various dosage forms. Among these CPM preparations, BCA is the most widely used with 10 preparations (62.50%), 5 preparations (31.25%) containing NBC, and one preparation (6.25%) containing BCS. According to our statistics and analysis, the clinical applications of BC in IS treatment mainly include 4 dosage forms, including 9 pills (56.25%), 4 tablets (25.00%), 2 capsules (12.50%), and one pulvis (6.25%) (Figure 6C). Overall, these insights provide valuable information about the prevalence of different types of BC in CPM preparations and their applications in the treatment of IS, along with the diverse dosage forms utilized in clinical practice.

### 6.5 Representative CPM preparations containing BC

Notably, three specific BC preparations targeting anti-IS are detailed in (Table 4), each providing essential information such as the preparation name, type, SFDA approval number, actions, and indications. Among these formulations, the noteworthy prescriptions incorporating NBC, BCA, and BCS are Ershiwuwei Zhenzhu Wan, Angong Niuhuang Pill, and Annao Pian, respectively (Chinese Pharmacopoeia Committee of People’s Republic of China, 2020; Peng et al., 2022). These meticulously crafted preparations hold significance in the field of traditional Chinese medicine, underscoring their role in combating IS as highlighted in the *Chinese Pharmacopoeia*.

**TABLE 4:** CPM preparations containing *Bovis Calculus* included in the *Chinese Pharmacopoeia* and commonly used to treat IS.

No.	CPM name	Type	SFDA approval number	Actions	Indications
1	Shixiang Fansheng Pill	NBC	Z12020754	To open orifices, resolve phlegm, tranquilize the mind with heavy medicinals	Apoplexy due to phlegm clouding the pericardium, manifested as delirious speech, coma, exuberant and congesting sputum, and trismus
2	Qishiwei Zhenzhu Pill	NBC	Z54020081	To tranquilize mind, unblock meridians, harmonize collaterals, harmonize qi and blood and open the orifices	Heimai and Baimai disease (Tibetan Medicine), Longxue disorders (Tibetan Medicine), apoplexy with paralysis or hemiplegia, epilepsy, cerebral haemorrhage, cerebral concussion, heart disease, hypertension and neurotic disorders
3	Renshen zaizao Pill	NBC	Z12020350	To tonify qi, nourish blood, dispel wind, resolve phlegm, activate blood and unblock the collaterals	Apoplexy with the pattern of qi deficiency with blood stasis and obstruction of collaterals by wind-phlegm, manifested as deviated eyes and mouth, hemiplegia, numbness of the limbs, pain, spasm, and sluggish speech
4	Angong Niuhuang Pill	NBC	Z21021326	Actions to clear heat, remove toxin, settle fright, and open the orifices	Febrile disease due to pathogenic factors entering the pericardium, manifested as high fever with seizure, loss of consciousness, and delirious speech; apoplex with coma, encephalitis, meningitis, toxic encephalopathy, cerebral haemorrhage, and septicemia with the symptoms described above
5	Angong Niuhuang Pill	NBC	Z11021084	To clear heat, remove toxin, settle fright and open the orifices	Febrile disease due to pathogenic factors entering the pericardium, manifested as high fever with seizure, loss of consciousness, and delirious speech; Apoplexy with coma, encephalitis, meningitis, toxic encephalopathy, cerebral hemorrhage, and septicemia with the symptoms described above
6	Tiandan Tongluo Tablet	BCA	Z20090152	To activate blood, unblock the collaterals, extinguish wind, and resolve phlegm	Apoplexy striking the meridians and collaterals with the pattern of wind-phlegm and blood stasis obstructing the meridians and collaterals, manifested as hemiplegia, hemianesthesia, deviated eyes and mouth, and sluggish speech; Acute stage and early stage of convalescence of cerebral infarction with the symptoms described above
7	Tiandan Tongluo Capsule	BCA	Z20010029	To activate blood, unblock the collaterals, extinguish wind, and resolve phlegm	Indications Apoplexy striking the meridians and collaterals with the pattern of wind-phlegm and blood stasis obstructing the meridians and collaterals, manifested as hemiplegia, hemianesthesia, deviated eyes and mouth, and sluggish speech; Acute stage and early stage of convalescence of cerebral infarction with the symptoms described above
8	Xinnaojing Tablet	BCA	Z21021359	To pacify the liver, subdue yang, clear the heart and tranquilize the mind	Dizziness and stroke (apoplexy) due to ascendant hyperactivity of liver yang, manifested as dizziness, blurred vision, vexation, restlessness, slurred speech, and paralyzed limbs; Hypertension with the symptoms described above
9	Annao Pill	BCA	Z23020126	To clear heat, remove toxin, tranquilize the mind and induce resuscitation, sweep phlegm, open the orifices, settle fright and extinguish wind	High fever, loss of consciousness, vexation, restlessness, delirious speech, convulsions, syncope, wind stroke with blocked orifices, headache and dizziness; Hypertension and cerebral apoplexy with the symptoms described above
10	Jiannao Anshen tablet	BCA	Z23020125	To clear heat, remove toxin, tranquilize the mind and induce resuscitation, sweep phlegm, open the orifices, settle fright and extinguish wind	High fever, loss of consciousness, vexation, restlessness, delirious speech, convulsions, syncope, wind stroke with blocked orifices, headache and dizziness; Hypertension and cerebral apoplexy with the symptoms described above
11	Qingxuan Zhitan Pill	BCA	Z11021187	To pacify the liver, extinguish wind, resolve phlegm and unblock the collaterals	Dizziness, vertigo, stiffness of the neck, distension in the head, heat in the chest, fear, deficiency vexation, exuberant and congesting phlegm, slobber, sluggish speech, numbness of the limbs, deviated eyes and mouth and hemiplegia due to ascendant hyperactivity of liver yang, and internal stirring of liver wind
12	Zaizao Wan	BCA	Z14021477	To dispel wind, resolve phlegm, activate blood and unblock the collaterals	Apoplexy due to wind phlegm obstructing the collaterals, manifested as hemiplegia, deviated tongue and mouth, numbness of the extremities, pain, spasm, and sluggish speech
13	Xueshuan Xinmaining Pill	BCA	Z20030145	To tonify qi, activate blood, open the orifices and relieve pain	Stroke and chest bi disorder due to qi deficiency with blood stasis, manifested as dizziness, vertigo, hemiplegia, oppression in the chest, heart pain, palpitations, and shortness of breath; Recovery phase of IS, coronary heart disease and angina pectoris with the symptoms described above
14	Xueshuan Xinmaining Capsule	BCA	Z22021474	To tonify qi, activate blood, open the orifices and relieve pain	Stroke and chest bi disorder due to qi deficiency with blood stasis, manifested as dizziness, vertigo, hemiplegia, oppression in the chest, heart pain, palpitations, and shortness of breath; Recovery phase of IS, coronary heart disease and angina pectoris with the symptoms described above
15	Kangshuan Zaizao Pill	BCA	Z21021307	To activate blood, resolve stasis, relax sinews, unblock the collaterals, extinguish wind and arrest convulsions	Wind stroke due to static blood obstructing orifices and failing to nourish meridians and collaterals, manifested as numbness of the limbs, difficulty in walking, paralysis, deviated eyes and mouth, slurred speech; Convalescence stage and sequela of apoplexy with the symptoms described above
16	Ershiwuwei Zhenzhu Pill	BCS	Z54020085	To tranquilize the mind and open the orifices	Apoplexy manifested as hemiplegia, deviated eyes and mouth, coma, disordered consciousness, delirious speech, and mania, etc

Abbreviations: BCS, *bovis calculus sativus;* BCA, *bovis calculus artifactus;* NBC, natural *bovis calculus;* CPM, chinese patent medicine; SFDA, state food and drug administration.

Ershiwuwei Zhenzhu Wan functions to decrease TNF-α, IL-1β, and IL-6 expression in the serum of pMCAO rats, thereby exerting anti-inflammation and antioxidation. Additionally, it enhances the activity of SOD and CAT while decreasing MDA levels. It participates in neuronal repair and regeneration by up-regulating Notch1, Jagged1, and Hes1. Moreover, Ershiwuwei Zhenzhu Wan inhibits neuronal apoptosis by up-regulating Bcl-2 mRNA and reducing the expression of caspase-3 protein. Specifically, in rats with CI, this treatment shows promise in increasing VEGF levels, promoting angiogenesis, and decreasing cerebral capillary permeability, brain water content, and brain edema. This may effectively prevent brain edema resulting from experimental CI in rats (Zhang et al., 2006; Liu et al., 2022). Different clinical research has demonstrated that Ershiwuwei Zhenzhu Wan may enhance the average velocity of blood flow in the brain, as well as improve blood circulation in the left and right vertebral arteries and the basilar artery. These improvements in hemodynamics help prevent blood stagnation, lower blood lipid levels, and reduce platelet aggregation and adhesion. Furthermore, it has the potential to enhance blood flow in microcirculation and exhibits a positive therapeutic impact on memory impairment resulting from cerebrovascular disease (Wang et al., 2013; Wang, 2021).

Studies demonstrated that Annao tablet has the ability to facilitate the transformation of astrocyte phenotype from A1 to A2 and microglia phenotype from M1 to M2 in the rat cortex, contributing to the maintenance of brain homeostasis and the enhancement of brain function after injury (Zhang, 2019). Annao tablet also improves mitochondrial fusion and mitotic disorders by increasing fusion and cleavage proteins Opa1 and Drp1, activating the PINK1/Parkin pathway, promoting mitochondrial autophagy in neurons, and timely removing damaged mitochondria. Additionally, Annao tablet has the potential to enhance the transcription level of Bcl-2 mRNA, augment the ratio of Bcl-2/Bax to impede apoptosis, diminish the count and protein expression of caspase-3 positive cells, decrease the number of neuronal apoptosis at the periphery of cerebral infarction, ameliorate the neurological deficit following ischemia, and elevate the count of positive neurons (Zhang Y. et al., 2020). Moreover, Annao tablet significantly enhances the cognitive abilities of rats with bilateral common carotid artery ischemia. It effectively elevates the concentration of acetylcholine in the hippocampus and mitigates neuronal apoptosis (Xu and Luan, 2016). Clinical trials in this century found Annao tablet is effective in improving acquired cognitive dysfunction syndrome and headache symptoms in patients with cerebral apoplexy (Yang and Luo, 2015).

Angong Niuhuang Pill exhibits anti-inflammatory properties by suppressing TNF-α, IL-1β, and iNOS mRNA expressing in the cerebral tissue of ischemic rats (Liu Y. X. et al., 2011; Yan et al., 2017). Angong Niuhuang Pill also diminishes MDA levels, enhances SOD activity, triggers the gsk-3β/HO-1 pathway activation, boosts the brain tissue’s resistance against oxidative damage (Feng and Sun, 2007; Zhang et al., 2021), and mitigates apoptosis by inhibiting the expression of Bax, Bcl-2, and caspase-3 (Wang et al., 2014). By reducing the water content of the tissue surrounding the cerebral hematoma, enhancing the deformability of red blood cells, improving brain edema, and inhibiting the levels of MMP9 mRNA and AQP4 mRNA in brain tissue, the integrity of the BBB is protected (Fang et al., 2007; Zheng et al., 2014; Li J. Z. et al., 2019; Chen et al., 2022). Furthermore, Angong Niuhuang Pill reduces overall blood thickness, plasma thickness, platelet clumping rate, red blood cell clumping and stiffness index, and red blood cell deformation index in the brain tissue of rats with CI (Liu Z. T. et al., 2011). Clinical studies have shown that Angong Niuhuang Pill can aid in managing high fever, promoting consciousness recovery, and reducing convulsions in patients with cerebral apoplexy. When combined with standard comprehensive treatment, it is effective in patients with cerebral apoplexy. Overall, Angong Niuhuang Pill has a significant advantage in the recovery of central nervous system function (Cui, 2012; Guo et al., 2014; Huang P. et al., 2018).

## 7 Pharmacokinetic characteristics of effective components

CA, CDCA, TDCA, and GDCA exhibit numerous therapeutic effects in IS. A comprehensive knowledge of their metabolic pathways is crucial in order to optimize the therapeutic effectiveness of BC. In the human intestinal Caco-2 cell model, a comparison was conducted to examine the uptake and transport of three types of BC. Furthermore, the bile acids in NBC were analyzed qualitatively and quantitatively using high-performance liquid chromatography-mass spectrometry (HPLC-MS). The results showed that GCA, CA, DCA, and TCA4 bile acids could be detected in brain tissue at different time points after intragastric administration, and the normalized area under curve (AUC) (0–60 min) was as follows: CA < GCA < DCA < TCA (Zhao et al., 2009). Moreover, ten blood entry components were found in the serum of rats after intragastric administration of NBC, and two known blood entry components were DCA and CDCA. Another study found that the pharmacokinetic characteristics of DCA and CDCA in rats were consistent with the two-compartment model (Han, 2012). After BCS and NBC intragastric administration in mice, CA, DCA, TCA, TDCA, CDCA, and Tau were detected in the blood. Additionally, BCS also detected GCA in both blood and tissues. Among them, the AUC (0–120 min) of CA, DCA, TCA, CDCA, and Tau was higher in the BCS group than in the NBC group (Feng, 2017).

The examination of the movement of various active substances in BC revealed the pharmacokinetic properties of its components. For instance, TCDCA in rats followed a first-order absorption one-compartment model. Additionally, it exhibited rapid absorption, gradual elimination, and prolonged duration in the body after being administered through the stomach (He, 2006). Furthermore, the concentrations of CA and HDCA in tissues were: kidney > lung > heart > brain. Among them, the target organs with a greater distribution were the lungs. The distribution of CDCA and DCA in the AUC and the heart, lungs, kidneys, and brain of mice was considerably higher compared to CA and HDCA. Noticeably, BR was observed to enhance bile acid absorption in BCA and reduce its elimination rate in animals, although it cannot elevate bile acid distribution in tissues. Study also foung that the oral bioavailability of monomeric bile acid is low, and the absorption and tissue distribution of bile acid in NBC is better than that in BCA (Zhao, 2007). Otherwise, after intraperitoneal injection of Qingkailing containing CA and HDCA, the peak time of CA in both normal and model groups (pMCAO model) was 4.8 min. The peak time of CA in the plasma of normal and model groups was 10.8 min and 12 min, respectively. This indicates that these two components entered the rat body quickly, and CI had no effect on its pharmacokinetic parameters (Liu X. et al., 2019).

Earlier studies have indicated that TCA concentration in serum, liver, kidney, and brain tissue of rats was determined following a single-dose intragastric administration of TCA. Additionally, it was found that the concentration-time curve of Tau in serum and liver conformed to the first-order absorption two-compartment model, while the concentration-time curve in kidney and brain tissue conformed to the first-order absorption one-compartment model. After oral administration, TCA exhibits rapid absorption, wide distribution, and slow elimination in rats (Hou, 2007). Besides, UDCA is a hydrophilic bile acid, which in consistent with the two-compartment model in rabbits. The drug is rapidly absorbed after entering the rabbit, and the peak concentration is reached in 5 min. Noticeably, the findings revealed that the majority of bile acids exhibited significantly reduced bi-directional permeability in BCA and BCS compared to NBC. Additionally, significant variations were observed in the excretion rates of CA, CDCA, TDCA, and GDCA among NBC and BCA groups, while no notable distinction was found between BCS and NBC groups (Chen et al., 2018).

Based on these findings, it can be inferred that the active constituents of BC have the ability to rapidly penetrate the bloodstream and reach the brain following absorption through the digestive system. This suggest that BC has the potential for the prompt management of stroke when taken orally.

## 8 Drug safety evaluation

### 8.1 Heavy metal problem

In recent years, the presence of heavy metals in BC and its preparations has been a concern for people. It is noteworthy that trace amounts of heavy metals, including Pb, Hg, and As, are often detected in BC (Takahashi et al., 2010). These heavy metals are typically absorbed from forage or the environment. Furthermore, BC from different sources may contain varying levels of heavy metals. Research indicates that NBC from Australia contains less As and Hg (Takahashi et al., 2010).

Importantly, the results indicating that heavy metal-containing BC preparations are not entirely absorbed after digestion by gastric fluid, with most being excreted from the body. Therefore, under proper medication guidance, the presence of heavy metals in BC and its preparations does not pose a significant risk to human health. To systematically evaluate the heavy metal problems, further research combining the absorption, metabolism, and excretion processes of heavy metals in the human body is necessary.

### 8.2 Drug allergy

Nowadays, BC and its related CPM preparations are widely used for medical purposes, indeed leading to some adverse events resembling allergic reactions. Studies indicated that adverse reactions caused by BC preparations (such as Niuhuang Jiedu Pill, Niuhuang Jiedu Tablet, Niuhuang Shangqing Pill, and Qingkailing Injection) mainly include allergic reactions, gastrointestinal disturbances (primarily diarrhea), and dysfunction of systems such as the nervous system (Huang et al., 2019). Additionally, allergic reactions to BC preparations are mainly characterized by rash, skin itching, and accompanied by systemic allergic reactions. Therefore, a more objective study is needed to evaluate the biosafety of BC, and it is recommended to use under the supervision of a healthcare professional.

#### 8.2.1 Acute toxicity

To date, BC and BC-related preparations have been widely used in IS treatment. According to a research, mice were divided into three groups in the laboratory, and after an 8-h fast, they were orally administered 12.5% BC at doses of 10 g/kg, 15 g/kg, and 20 g/kg, respectively. In a 7-day acute toxicity experiment, none of the groups exhibited signs of toxicity, with normal eating and bowel movements observed (Yuan et al., 1992). Currently, data on *in vivo* and clinical toxicity experiments of BC are still lacking. Therefore, it is necessary to conduct *in vivo* and clinical research on the potential toxicity of BC, which will be crucial to determine its safe dosage.

### 8.3 Potential side effects of drug interactions

When BC or its alternative preparations are used concomitantly with other Chinese herbal medicines or chemical substances, they generally do not induce or exacerbate side effects. Furthermore, studies have indicated that BC may reduce the incidence of adverse reactions to other drugs. For instance, BCS can reduce the incidence of Fecal occult blood in patients with acute IS and disturbance of consciousness treated with Zhongfeng Xingnao Liquid (Chen, 2022). Other research has reported that the co-administration of BCS with Gastrodin Injection reduced the incidence of adverse reactions in acute IS patients from 15.63% to 6.25% (Liao, 2020). Noticeably, novel drug delivery system has demonstrated good therapeutic efficacy, with researchers developing a new mucoadhesive film combining BCS with Ornidazole (OD) for oral ulcers treatment. This CBS-OD mucoadhesive film can reduce the adverse effects associated with conventional OD membranes, including significantly alleviating mucosal damage (Li W. et al., 2016).

## 9 Ethics of *Bovis Calculus*

The naturally occurring BC was first discovered by ancient Chinese ancestors while slaughtering sick cattle. Through clinical research and practice, they gradually realized the medicinal value of BC and began using it in clinical settings. From the perspective of sources, NBC forms naturally in cattle during the course of digestive or hepatobiliary diseases. Butchers intentionally search for NBC when inspecting the liver and gallbladder of cattle. This method is fundamentally different from the controversial practice of extracting bile from live bears, ensuring that NBC fully aligns with humanitarian and medical ethics.

Considering the scarcity and high price of NBC, it is important to develop its substitutes (Li C. et al., 2020). In the 1970s, Chinese researchers successfully developed the CBC, and the Ministry of Drug Administration approved three substitutes for NBC, namely, BCS, BCA, and CBC (Cai, 1978; Qiao et al., 2011; Shimada et al., 2013). However, due to the high cost, long production cycle, and ethical concerns in the medical field, CBC has limited circulation in the market and has not been included in the *Chinese Pharmacopoeia* (Liu et al., 2019a). The analysis and discussion presented in this study fall under the categories of NBC, BCA, and BCS, all of which adhere to the medical ethics of BC and the collection standards of the *Chinese Pharmacopoeia*.

## 10 Discussions and outlooks

### 10.1 Active ingredients

Clinical trials in the past found that IS injury involves complex pathophysiological processes. Reviewing the literature reveals that BC and its active components offer advantages in improving microcirculation disturbance, reducing excitatory amino acid toxicity, inhibiting oxidative stress, protecting the BBB, preventing apoptosis, and promoting nerve and vascular regeneration. Further analysis revealed that the primary anti-excitatory amino acid toxic components of BC were TUDCA and Tau. The main components that counteract oxidative stress injury are BV, BR, CA, TUDCA, HDCA, and Tau. Moreover, the main anti-inflammatory components were CA, UDCA, TUDCA, HDCA, and Tau. Besides, the main components protecting the BBB function were GUDCA, CA, TUDCA, and UDCA. Furthermore, the primary anti-apoptosis components included CA, Tau, TUDCA, BV, and BR.

### 10.2 The correlation analysis of composition and therapeutic effect

BCA and BCS are substitutes for NBC. According to the existing literature, it is found that the main active components of the three types of BC are bile acids, bile pigments, Tau, amino acids, and a variety of trace elements. Among them, Tau, BR, BV, HDCA, CA, HDCA, and TUDCA have been widely studied in the research and application of IS. While NBC and its substitutes have been extensively utilized in clinical settings, NBC is acknowledged to possess superior effectiveness in comparison to its surrogates. This is possibly attributed to the varying proportions of the constituents found in NBC and its substitutes (Yan et al., 2007).

BCA is one of the main substitutes for NBC, and there are some differences and similarities between them in composition. For example, the content and proportion of bile acids in BCA are prepared based on the proportion of NBC. The bile acid content in BCA is higher than that in NBC, but other non-bile acid components are lower in BCA compared to NBC. These components include BR, inorganic ions, amino acids, and so on. It is noteworthy that BR is the most abundant component in NBC, constituting 35%–60% of its content. NBC contains substances that are absent in BCA, which can enhance the absorption and distribution of bile acids in BCA. In addition, BR can enhance the absorption of bile acid and reduce its elimination rate in mice (Zhao, 2007). Moreover, as a substitute for NBC, BCS has been included in the *Chinese Pharmacopoeia* and can replace NBC in clinical practice. This suggests that in a wider range of treatment scenarios, such as remote areas where medical personnel and equipment are less developed, the lower price of BCS can replace NBC to some extent. The analysis of the content properties of Tau and 12 bile acids in BCS and NBC revealed that the concentration of CA/DCA in BCS was approximately double that in NBC. Furthermore, the ratio of unbound bile acids to bound bile acids was higher in NBC compared to BCS. However, there was no notable disparity in the Tau/(TCA + TDCA + TCDCA) content between BCS and NBC (Feng, 2017). The aforementioned findings indicate that there are certain resemblances and disparities in the chemical makeup and substance of the three types of BC, which may be associated with their differences in pharmacodynamics.

### 10.3 Current research challenges and future research directions

The current research on BC and its pharmacological effects in IS faces several limitations and challenges. Many studies have concentrated on the mechanism of BC intervention in CI/RI. However, the following issues persist:

1) There is confusion around the terminology “*Bovis Calculus*”. *Bovis Calculus* is clearly defined in the *Chinese Pharmacopoeia*, but in commercial trade or scientific research, its Chinese pronunciation is very similar to that of another TCM, “*Sulfur*”. However, the pharmacological actions of these two substances are completely different, as *Sulfur* is a mineral extracted from natural sulfur-containing ores.
2) Lack of comprehensive and well-controlled clinical trials to evaluate the efficacy and safety of BC in the treatment of IS. Many existing studies have small sample sizes, use limited evaluation methods, and lack quality, making the reliability of their conclusions questionable.
3) Inconsistencies in the doses of BC and its preparations used by different research institutions and researchers, pose new challenges in deriving meaningful comparative results and conclusions.
4) Most of the existing literature focuses on the anti-IS damage effects of some active components in BC, but there are few comparative studies on the anti-CI effects of single BC and different sources of BC. The mechanisms of other active components, such as cholesterol components, in BC against IS injury and their relationship with the main active components of BC are not clear.
5) NBC substitutes are widely used in clinics, and it is believed that there are variations in therapeutic effects among these substitutes as well as compared to NBC, but the underlying reasons for these variances have not been elucidated. Whether the combined components have better therapeutic effects than the individual ones still requires further study.
6) The exact mechanisms of action of BC in treating IS are not fully elucidated. Although some studies have shown that BC can improve hemorheological parameters, alleviate inflammatory responses, and enhance antioxidant capacity, the precise molecular mechanisms remain unclear.
7) According to the location of CI, IS can be categorized into focal CI and global CI. It can also be classified as permanent CI or ischemia-reperfusion based on whether reperfusion occurs after the ischemic event. However, most researchers mainly focus on the focal IS model. Due to the treatment time window and other factors, more patients experience permanent CI (Dalli et al., 2019). It is advocated to verify the neuroprotective effect of BC using a variety of CI models. It is suggested that the timing and method of administration in the model should align with clinical practice.
8) The restoration stage of CI has a significant effect on the quality of life of patients. At present, research on the anti-IS effects of BC and its active components is primarily concentrated on alleviating IS injury in the acute stage, with insufficient focus on the restoration stage post-IS. Future research should pay attention to the follow-up period.
9) Patients often develop a fever after undergoing IS. Long-term fever has adverse effects on the prognosis of patients. Tau and bile acids in BC also play an anticoagulant and antipyretic role in the process of CI (Zhang et al., 2013; Xu, 2015). The mechanisms of these actions need to be further explored.
10) There is a lack of research on the potential chronic toxicity and hypersensitivity reactions of BC, as well as a lack of studies on the interchangeability of NBC with other substances like BCA and BCS in clinical applications.

In response to the limitations and challenges in the pharmacological treatment of IS using BC, we propose the following research directions:

1) The bioactive compounds, potential targets, and potential mechanisms of BC were studied using the method of network pharmacology. It was found that the PI3K/AKT and MAPK signal pathways were the key targets of BC against IS (Liu et al., 2021). BC in the treatment of IS is associated with steroid hormone biosynthesis, metabolic pathways, and neuroactive ligand-receptor interactions (Chen et al., 2021). Furthermore, network pharmacology combined with cell experiments further confirmed that BCS can protect against OGD/R damage through anti-apoptotic effects, maintaining BBB tight junction proteins, anti-inflammatory actions, and inhibiting oxidative stress. BCS also protects NVU by regulating the HIF-1/VEGF and PI3K/Akt signaling pathways (Du et al., 2022). The conclusion of network pharmacology is only a conjecture based on big data. It is also limited by the selected database. Therefore, further verification is needed to determine if BC interferes with IS through other pathways.
2) Conduct larger-scale and more standardized pharmacological experiments and clinical trials to actively evaluate the efficacy and safety of BC in IS treatment. Establish standard specifications for BC and its preparations to ensure the reliability of the research.
3) Further in-depth research is needed to elucidate the molecular mechanisms of how BC exerts its therapeutic effects on IS. The research aims should focus on clarifying how BC improves blood flow, reduces inflammation, and enhances antioxidant activity, providing a solid scientific foundation for its clinical applications.
4) Efforts should be made to address the issue of the confusing nomenclature of BC products, ensuring consistency between commercial circulation and scientific research. Emphasis should be placed on using “*Bovis Calculus*” as the specific identifier that complies with the *Chinese Pharmacopoeia.*
5) Investigations should be conducted to explore the potential causes of adverse reactions associated with the use of BC, and to study the interchangeability of artificial substitutes in clinical applications. Through the discussion of these research directions, we can further deepen the understanding and utilization of BC in IS treatment.

## 11 Conclusion

This research highlights the tremendous potential of BC in the prevention and treatment of IS through a multi-component, multi-target, multi-pathway approach. Comprehensive studies have shown that BC can treat CI/RI through mechanisms such as improving microcirculation disturbances, inhibiting neurotoxicity, reducing ROS damage, alleviating inflammatory responses, suppressing cell apoptosis, and reducing ischemic brain tissue damage. Furthermore, BC has the ability to mitigate BBB injury and prevent calcium overload caused by CI. It can also promote angiogenesis and functional recovery of the brain during the post-stroke rehabilitation period. Additionally, BC exhibits anti-thrombotic effects by inhibiting platelet aggregation and fibrinolysis, and exerts anti-CI/RI effects by improving lipid metabolism and regulating the PI3K/AKT and MAPK signaling pathways. Therefore, BC not only can alleviate the damage caused by ischemia to the brain, but also has significant interventional effects on related complications and sequelae. Besides, Chinese patent medicine formulations containing BC are clinically widely used in the treatment of IS, which offers a new and promising avenue for IS. In summary, BC has notable advantages and tremendous development potential in the prevention and treatment of IS. Future research should delve deeper into exploring its broader biological activities and clinical application value.

## Glossary

8-OHdG: 8-hydroxy-2′-deoxyguanosine
A.D.: Anno domini
AA: Arachidonic acid
ADP: Adenosine diphosphate
Asp: Aspartic acid
AUC: Area under curve
B.C.: Before christ
BBB: Blood-brain barrier
BDNF: Brain-derived neurotrophic factor
BR: Bilirubin
BV: Biliverdin
CA: Cholic acid
cAMP: Cyclic adenosine monophosphate
BCA: *Bovis Calculus artifactus*
CBF: Cerebral blood flow
BCS: *Bovis Calculus sativus*
CBC: Cultured *Bovis Calculus*
CDCA: Chenodeoxycholic acid
CI/RI: Cerebral ischemia/reperfusion injury
CPM: Chinese patent medicine
CRS-R: Coma Recovery Scale-Revised
CytC: Cytochrome C
DCA: Deoxycholic acid
EAA: Excitatory amino acids
ESR: Erythrocyte sedimentation rate
FIB: Fibrinogen
FOUR: Full Outline of UnResponsiveness
GABA: γ-aminobutyric acid
**GABA**_**A**_ **R**: γ-aminobutyric acid type a receptors
GCA: Glycocholic acid
GCDCA: Glycochenodeoxycholic acid
GCS: Glasgow Coma Scale
GDCA: Glycodeoxycholic acid
GDNF: Glia cell line-derived neurotrophic factor
Glu: Glutamic acid
Gly: Glycine
GSH: Glutathione
GSH-Px: Glutathione peroxidase
GUDCA: Glycoursodeoxycholic acid
HCT: Hematocrit
hs-CRP: High-sensitivity c-reactive protein
IAA: Inhibitory amino acids
ICAM-1: Intercellular adhesion molecule-1
IL-1β: Interleukin-1β
IL-6: Interleukin-6
IL-8: Interleukin-8
iNOS: Inducible nitric oxide synthase
IS: Ischemic stroke
LDL-C: Low-density lipoprotein cholesterol
MCP-1: Monocyte chemoattractant protein-1
MDA: Malondialdehyde
MMP-9: Matrix metalloproteinase-9
mRS: Modified Rankin Scale
NADPH: Nicotinamide adenine dinucleotide phosphate
NBC: Natural *Bovis Calculus*
NF-κB: Nuclear factor-κb
NIHSS: National Institute of Healthstroke scale
NMDA: N-methyl-D-aspartate
NO: Nitric oxide
NSE: Neuron specific enolase
NVU: Neurovascular unit
OD: Ornidazole
OGD/R: Oxygen-glucose deprivation/reperfusion
ROS: Reactive oxygen species
rt-PA: Recombinant tissue-type plasminogen activator
SOD: Superoxide dismutase
T-AOC: Total anti-oxidative capability
Tau: Taurine
TCA: Taurocholic acid
TCDCA: Taurochenodeoxycholic acid
TDCA: Taurodeoxycholic acid
TEER: Transepithelial electrical resistance
TG: Triglycerides
TGR5: Takeda g-protein receptor 5
TNF-α: Tumor necrosis factor-α
TUDCA: Tauroursodeoxycholic acid
UDCA: Ursodeoxycholic acid
VCAM-1: Vascular cell adhesion molecule-1
VEGF: Vascular endothelial growth factor
WBV: Whole blood viscosity
ZO-1: Zonula occludens-1
γ-GT: Gamma-glutamyl transferase
